# Binding interface between the Salmonella σ^S^/RpoS subunit of RNA polymerase and Crl: hints from bacterial species lacking *crl*

**DOI:** 10.1038/srep13564

**Published:** 2015-09-04

**Authors:** Paola Cavaliere, Christina Sizun, Fabienne Levi-Acobas, Mireille Nowakowski, Véronique Monteil, François Bontems, Jacques Bellalou, Claudine Mayer, Françoise Norel

**Affiliations:** 1 Institut Pasteur, Laboratoire Systèmes Macromoléculaires et Signalisation, Département de Microbiologie, 25 rue du Docteur Roux, 75015 Paris, France; 2 CNRS ERL3526, rue du Docteur Roux, 75015 Paris, France; 3 Institut de Chimie des Substances Naturelles, CNRS UPR2301, 91190 Gif-sur-Yvette, France; 4 Institut Pasteur, Plate-forme de Protéines Recombinantes, Département de Biologie Structurale et Chimie, 25 rue du Docteur Roux, 75015 Paris, France; 5 CNRS UMR 3528, rue du Dr. Roux, 75015 Paris, France; 6 Institut Pasteur, Unité de Microbiologie Structurale, Département de Biologie Structurale et Chimie, 25 rue du Docteur Roux, 75015 Paris, France; 7 Université Paris Diderot, Sorbonne Paris Cité, Paris, France

## Abstract

In many Gram-negative bacteria, including *Salmonella enterica* serovar Typhimurium (*S*. Typhimurium), the sigma factor RpoS/σ^S^ accumulates during stationary phase of growth, and associates with the core RNA polymerase enzyme (E) to promote transcription initiation of genes involved in general stress resistance and starvation survival. Whereas σ factors are usually inactivated upon interaction with anti-σ proteins, σ^S^ binding to the Crl protein increases σ^S^ activity by favouring its association to E. Taking advantage of evolution of the σ^S^ sequence in bacterial species that do not contain a *crl* gene, like *Pseudomonas aeruginosa*, we identified and assigned a critical arginine residue in σ^S^ to the *S*. Typhimurium σ^S^-Crl binding interface. We solved the solution structure of *S.* Typhimurium Crl by NMR and used it for NMR binding assays with σ^S^ and to generate *in silico* models of the σ^S^-Crl complex constrained by mutational analysis. The σ^S^-Crl models suggest that the identified arginine in σ^S^ interacts with an aspartate of Crl that is required for σ^S^ binding and is located inside a cavity enclosed by flexible loops, which also contribute to the interface. This study provides the basis for further structural investigation of the σ^S^-Crl complex.

In bacteria, a primary housekeeping sigma factor and one or more alternative σ factors associate with the catalytically active RNA polymerase (RNAP) core enzyme (α2ββ’ω, E), to form the holoenzyme Eσ, and direct transcription initiation of specific subsets of genes^1^,^2^. In many Gram-negative bacteria, σ^S^/RpoS is produced during late exponential phase, or in response to stress, to modify global gene transcription and to allow stationary phase survival and general stress resistance^3^,^4^,^5^. In the wide host-range pathogen *S*. Typhimurium, σ^S^ is not only required for general stress resistance, but also for virulence, biofilm formation and development of the red dry and rough (rdar) morphotype, a colony morphology caused by the production of amyloid fibers (curli) and cellulose^6^,^7^,^8^.

The efficiency of formation of the housekeeping and alternative Eσ can be modulated by regulatory factors that bind E and/or σ^5^,^9^. So far, Crl is the only known σ^S^-dedicated regulatory factor that enhances σ^S^ activity through a direct interaction, favouring Eσ^S^ formation^7^,^10^,^11^,^12^,^13^,^14^,^15^. Analyses of sequenced bacterial genomes revealed that *crl* is less widespread and less conserved at the sequence level than *rpoS*^16^. Nevertheless, Crl family members perform the same biological function and share a common mechanism of σ^S^ binding^17^,^18^. Moreover, the X-ray crystal structure of Crl from *Proteus mirabilis* (Crl_PM_) and mutational analyses strongly suggest that σ^S^ binds to a Crl cavity enclosed by flexible loops^16^,^17^,^18^. In contrast to housekeeping σ factors, a three dimensional structure is not available for σ^S^. However, sequence conservation between σ^S^ and housekeeping σ suggests that, like housekeeping sigma factors, σ^S^ contains four structural domains connected by flexible linkers^19^,^20^. Domain 2 (σ_2_), the most highly conserved domain of σ factors^19^,^20^, is the only σ^S^ domain involved in Crl binding, and two regions of σ^S^_2_ are required for interaction^16^,^21^. In the structural model of *S.* Typhimurium σ^S^ (σ^S^_STM_)^16^, these two regions are accessible and close together, consisting of an α-helix (α2) and the DPE motif located within a long loop just on top of helix α2 (Fig. 1a,b).

Many bacterial species containing *rpoS* do not harbour *crl* in their genome, such as *Pseudomonas aeruginosa*^22^, in which σ^S^ is involved in stress resistance and in the production of virulence factors^8^. We show here that *Pseudomonas aeruginosa* σ^S^ (σ^S^_PA_) does not interact with Crl, despites the conservation of the DPE motif. Most interestingly, substitution of one single residue in the helix α2 of σ^S^_PA_ was sufficient to confer to σ^S^_PA_ the ability to bind Crl, and our data assigned this residue to the σ^S^-Crl binding interface. By NMR, we solved the solution structure of *S.* Typhimurium Crl (Crl_STM_) and used it for NMR binding assays with σ^S^_STM._ Furthermore, *in silico* models of the σ^S^_STM_-Crl_STM_ complex were generated based on mutational analyses. The output models show that two specific salt bridges can be formed between Crl and σ^S^, in agreement with our previous biophysical data suggesting that σ^S^-Crl complex formation is driven by electrostatic interactions^18^.

## Results

### Crl does not activate σ^S^ from *Pseudomonas aeruginosa*

**Figure 1:**
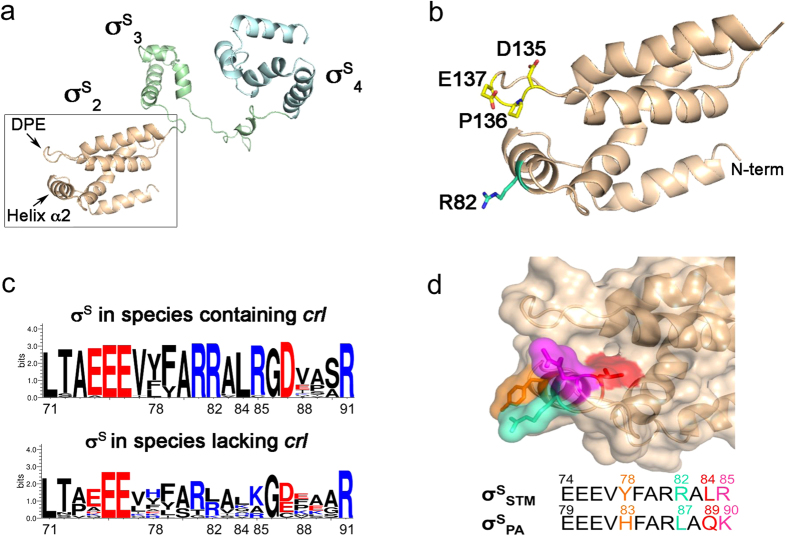
The Crl binding region of *S.* Typhimurium σ^S^ and sequence comparison of σ^S^ from species harbouring *crl* or lacking *crl.* (**a**) Cartoon representation of the structural model^16^ of full-length σ^S^_STM_, in which domains σ^S^_2_, σ^S^_3_ and σ^S^_4_ are shown. The helix α2^16^,^21^ and the DPE motif^21^ within σ^S^_2_ are highlighted. (**b**) Zoomed view of σ^S^_2_ in which the side chains of critical residues are depicted in cyan and yellow. (**c**) WebLogo (http://weblogo.threeplusone.com/create.cgi) of σ^S^ residues 71–91 (numbered as in σ^S^_STM_) generated with the σ^S^ sequences listed in Supplementary Figure S2, from bacterial genomes containing *crl* or lacking *crl*. (**d**) Sequence alignment of σ^S^ helix α2 from *S.* Typhimurium and *P. aeruginosa*, both with their own numbering. Residues that differ between σ^S^_PA_ and σ^S^_STM_ are highlighted in the alignment and represented with the same colour code as in the surface representation of *S.* Typhimurium σ^S^_2_ shown above.

σ^S^_2_ is the only domain involved in the interaction with Crl^16^,^18^,^21^ and two regions, close together on the structural model of σ^S^_STM_^16^ (Fig. 1a,b), were identified as the Crl binding regions: the helix α2, corresponding to residues 74 to 85 in σ^S^_STM_^16^,^21^, and the DPE motif, corresponding to residues 135 to 137 and initially identified in σ^S^ from *E. coli*^21^. Consistently, a fragment of σ^S^_STM_ domain 2 lacking this motif, σ^S^_STM (1–136)_, and σ^S^_STM_ variants at position D135 and E137, were not able to interact with Crl in bacterial two hybrid (BACTH) assays (Supplementary Fig. S1), confirming that the DPE motif in σ^S^_STM_ is involved in Crl binding.

**Figure 2:**
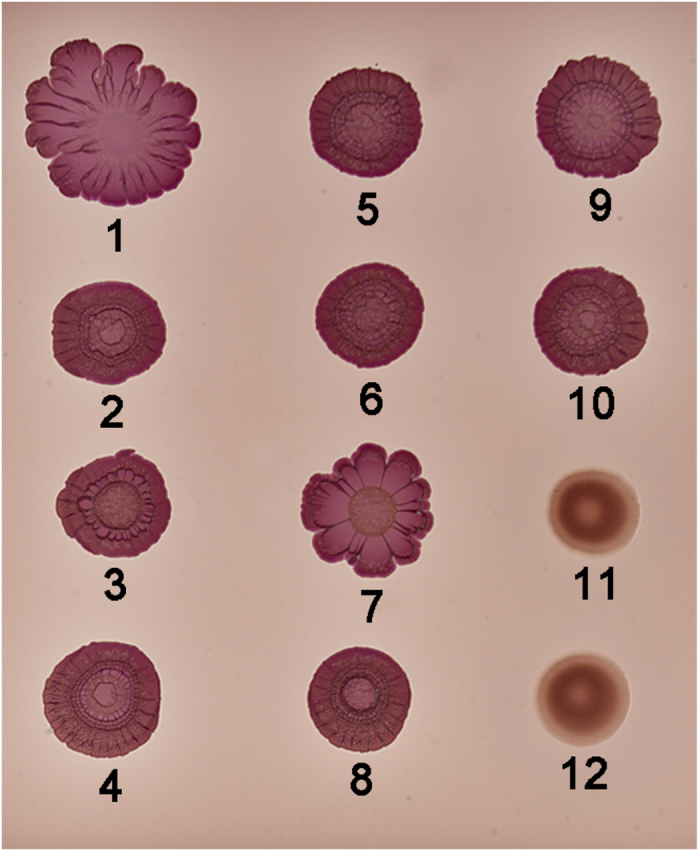
*In vivo* activity of σ^S^ variants and their sensitivity to Crl activation. Development of the red dry and rough (rdar) morphotype by *S.* Typhimurium strains harbouring wild-type and mutant *rpoS* alleles and the effect of a Δ*crl* mutation: spot 1, wild-type strain ATCC14028; spot 2, ATCC14028 Δ*crl*; spot 3, ATCC14028 *rpoS*_PA_; spot 4, ATCC14028 *rpoS*_PA_ Δ*crl*; spot 5, ATCC14028 *rpoS*_STM-R82L_; spot 6, ATCC14028 *rpoS*_STM-R82L_ Δ*crl*; spot 7, ATCC14028 *rpoS*_PA-L87R_; spot 8, ATCC14028 *rpoS*_PA-L87R_ Δ*crl*; spot 9, ATCC14028 *rpoS*_STM-R82E_; spot 10, ATCC14028 *rpoS*_STM-R82E_ Δ*crl*; spot 11, ATCC14028 Δ*rpoS*; spot 12, ATCC14028 Δ*rpoS* Δ*crl*.

**Figure 3:**
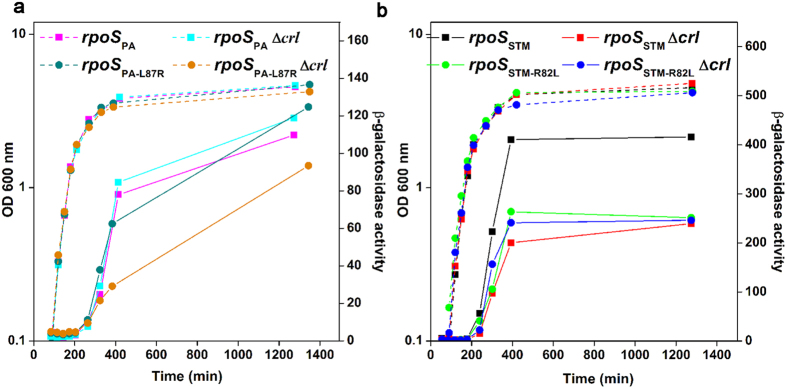
Expression kinetics of the *katN-lacZ* transcriptional fusion in *S*. Typhimurium strains harbouring different *rpoS* alleles. Growth (dashed lines) and β-galactosidase activities (solid lines) of the *S*. Typhimurium strains indicated, harbouring wild-type and mutant *rpoS* alleles from *P. aeruginosa* (**a**) and *S*. Typhimurium (**b**). Aliquots were taken at different time intervals during growth and β-galactosidase activity was measured in Miller units. Aliquots were also used for σ^S^ immunodetection (Supplementary Fig. S3b). The growth phase was determined by the measurement of culture turbidity at OD 600 nm. The experiments were repeated twice with similar results.

However, the DPE motif is conserved in σ^S^ of *P. aeruginosa* (σ^S^_PA_) that does not have *crl*^22^, and the sequence of the helix α2 differs from σ^S^_STM_ by only four residues (Fig. 1c,d and Supplementary Fig. S2), prompting us to examine whether σ^S^_PA_ can be activated by Crl. σ^S^_PA_ activity and its response to Crl were evaluated in a *S.* Typhimurium strain in which the native *rpoS* gene was replaced by the *rpoS* allele from *P. aeruginosa* (*rpoS*_PA_). Development of the rdar morphotype of *S.* Typhimurium is highly dependent on σ^S^ and Crl^7^ (compare spots 1, 2 and 11, Fig. 2). The *S*. Typhimurium strain harbouring the *rpoS*_PA_ allele was able to develop the rdar morphotype, in contrast to the Δ*rpoS* mutant of *S.* Typhimurium (compare spots 3 and 11, Fig. 2), indicating that σ^S^_PA_ was expressed and functional in this strain. However, the rdar morphotype of the *rpoS*_PA_ strain was similar to that of the *S.* Typhimurium Δ*crl* mutant and was not affected by a Δ*crl* mutation (compare spots 2, 3 and 4, Fig. 2), suggesting that σ^S^_PA_ did not respond to Crl. We also followed the expression of the *rpoS*-dependent *katN*-*lacZ* transcriptional fusion^13^ and of the σ^S^ protein, in wild type and Δ*crl S.* Typhimurium strains harbouring the *rpoS*_STM_ and *rpoS*_PA_ alleles (Fig. 3 and Supplementary Fig. S3). The growth kinetics of strains harbouring *rpoS*_STM_ and *rpoS*_PA_ were similar and expression of *katN-lacZ* and σ^S^ was induced in stationary phase, as expected^13^. The lower expression level of *katN-lacZ* in the *rpoS*_PA_ strain, compared to that in the *rpoS*_STM_ strain, could be due to differences in the expression level or intrinsic activities of σ^S^_PA_ with respect to σ^S^_STM_. Activation of *katN-lacZ* expression by Crl in *Salmonella* harbouring the *rpoS*_STM_ allele was maximal at the entry to stationary phase when σ^S^ begins to accumulate (Fig. 3b), as previously reported^13^. Indeed, at the entry to stationary phase, *katN-lacZ* expression was decreased and delayed by the Δ*crl* mutation. In contrast, no significant effect of the Δ*crl* mutation on *katN-lacZ* expression was detected in *Salmonella* containing the *rpoS*_PA_ allele (Fig. 3a), even though Crl amounts were similar in the *rpoS*_PA_ and *rpoS*_STM_ strains (Supplementary Fig. S3c).

**Figure 4:**
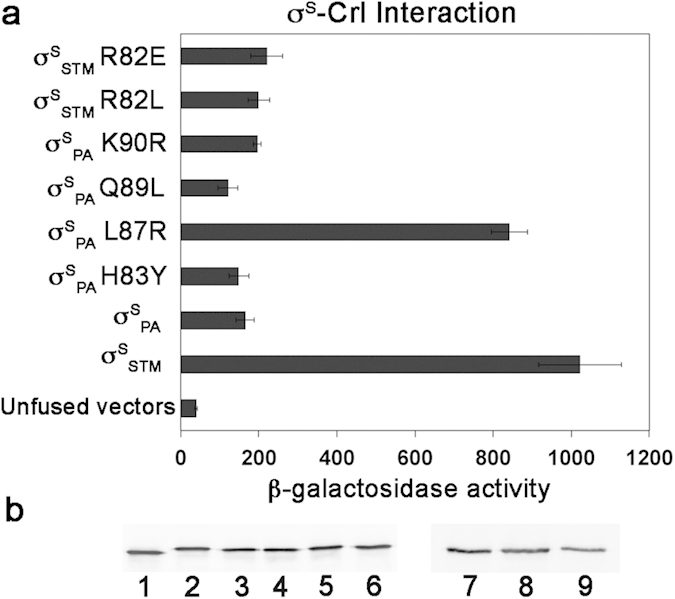
BACTH interaction analyses between Crl from *S*. Typhimurium and σ^S^ wild-type and variant proteins from *S.* Typhimurium and *P. aeruginosa*. (**a**) Interaction between Crl_STM_-T18 and the T25-σ^S^ hybrid proteins indicated was quantified by measuring β-galactosidase activity in Miller units. Results are the mean of at least three independent experiments and standard deviations are indicated with black bars. (**b**) Immunodetection of T25-σ^S^ fusion proteins by antibodies directed against the T25 polypeptide. Lane 1, σ^S^_STM_; lane 2, σ^S^_PA_; lane 3, σ^S^_PA_ H83Y; lane 4, σ^S^_PA_ L87R; lane 5, σ^S^_PA_ Q89L; lane 6, σ^S^_PA_ K90R; lane 7, σ^S^_STM_; lane 8, σ^S^_STM_ R82L; lane 9, σ^S^_STM_ R82E.

To determine whether the failure of σ^S^_PA_ to respond to Crl activation resulted from a lack of interaction between the two proteins, we used the BACTH *in vivo* assay (Fig. 4) and isothermal titration calorimetry (ITC) *in vitro* assay (Supplementary Fig. S4a). Unlike σ^S^_STM_^16^,^18^, σ^S^_PA_ did not interact with Crl, suggesting that unidentified σ^S^_STM_ residues, not conserved in σ^S^_PA_, are crucial for Crl binding.

### A single amino acid substitution renders σ^S^_PA_ sensitive to Crl activation

To further refine our understanding of σ^S^_2_ residues involved in Crl binding, the amino acid sequence of σ^S^_2_ was compared in bacterial species harbouring *crl* in their genome and in those lacking *crl* (Fig. 1c and Supplementary Fig. S2). Whereas the DPE motif was well conserved, the sequence of the helix α2 was more variable, especially in σ^S^ from strains lacking *crl*. In this region, the sequence between σ^S^_STM_ and σ^S^_PA_ differs by four surface-exposed residues (Y78, R82, L84 and R85 in σ^S^_STM_ that correspond to H83, L87, Q89 and K90 in σ^S^_PA_, respectively) (Fig. 1d). Two of these (R82 and L84 in σ^S^_STM_) are conserved in all σ^S^ proteins from species harbouring *crl*, but less conserved in σ^S^ from species lacking *crl.* To determine whether the non-conserved residues at position 83, 87, 89 and 90 in σ^S^_PA_ were responsible for the defect in Crl binding, we constructed σ^S^_PA_ variants in which the σ^S^_STM_ sequence was restored at these positions, and assessed their ability to interact with Crl in the BACTH assay (Fig. 4). Expression levels of σ^S^_PA_ wild type and variants were similar (Fig. 4b). Interestingly, one variant, σ^S^_PA_ L87R, was able to interact with Crl (Fig. 4a), suggesting that an arginine at position 87 in σ^S^_PA_ (corresponding to position 82 in σ^S^_STM_) is of paramount importance for Crl binding. This finding was further confirmed *in vitro* by ITC (Supplementary Fig. S4c,d). Interestingly, σ^S^_PA_ L87R and wild-type σ^S^_STM_ showed similar affinity for Crl (Supplementary Table S1). The major difference observed between σ^S^_PA_ L87R and σ^S^_STM_ was in the value of Δ_b_H, which was less negative for σ^S^_PA_ L87R than for σ^S^_STM_. This suggests that the number and type of intermolecular interactions in Crl-σ^S^_PA_ L87R and Crl-σ^S^_STM_ complexes might be slightly different, due to non-conserved σ^S^ residues affecting directly or indirectly the σ^S^-Crl binding interface. However, the Δ_b_S and Δ_b_G values were similar for σ^S^_PA_ L87R and σ^S^_STM_, endorsing the key role of an arginine at position 87 in the σ^S^_PA_ variant.

To assess whether the interaction between σ^S^_PA_ L87R and Crl was functional (*i.e.* whether Crl activates σ^S^_PA_ L87R), we monitored the rdar morphotype of *S.* Typhimurium harbouring the chromosomal *rpoS*_PA-L87R_ allele expressing σ^S^_PA_ L87R. Morphotypes of the *rpoS*_PA-L87R_ and wild type *Salmonella* strains were similar and dependent on *crl* (compare spots 1, 7 and 2, 8, Fig. 2), suggesting that σ^S^_PA_ L87R was able to respond to Crl activation. Consistently, *katN-lacZ* expression level in the *rpoS*_PA-L87R_ strain was decreased by the Δ*crl* mutation (Fig. 3a). Altogether these results demonstrated that substitution L87R renders σ^S^_PA_ sensitive to Crl activation.

### Residue R82 in σ^S^_STM_ is required for Crl binding and activation

To evaluate the impact in Crl binding of σ^S^_STM_ residue R82, corresponding to L87 in σ^S^_PA_, (Fig. 1d), the ability of the σ^S^_STM_ R82L variant to interact with Crl was assessed in BACTH and ITC assays (Fig. 4 and Supplementary Fig. S4b). Both assays showed that σ^S^_STM_ R82L does not interact with Crl. Consistently, development of the rdar morphotype and *katN-lacZ* expression levels were similar in the *rpoS*_STM-R82L_ strain (whatever its *crl* status) and the Δ*crl* strain harbouring the wild-type *rpoS*_STM_ allele (spots 5, 6 and 2, Fig. 2 and Fig. 3b), indicating that σ^S^_STM_ R82L was not activated by Crl. Far-UV CD spectra showed a similar secondary and tertiary structure for σ^S^_STM_, σ^S^_STM_ R82L, σ^S^_PA_ and σ^S^_PA_ L87R (Supplementary Fig. S4e,f), indicating that the σ^S^ conformation was similar in the four proteins. In the absence of Crl, *katN-lacZ* expression level was similar in the *rpoS*_STM-R82L_ and *rpoS*_STM_ strains (Fig. 3b), suggesting that σ^S^ stability and its interaction with the core RNAP were not affected by the R82L substitution. To assess the effects of a more drastic amino acid substitution at position 82, the σ^S^_STM_ R82E variant was characterized. Expression level and activity of this variant were similar to those of σ^S^_STM_ R82L (spots 9, 10 and 5, 6, Fig. 2 and Supplementary Fig. S5). Altogether, these findings suggested that σ^S^_STM_ R82 plays a key role in Crl binding and activation.

### Solution structure of *Salmonella* Crl

**Figure 5:**
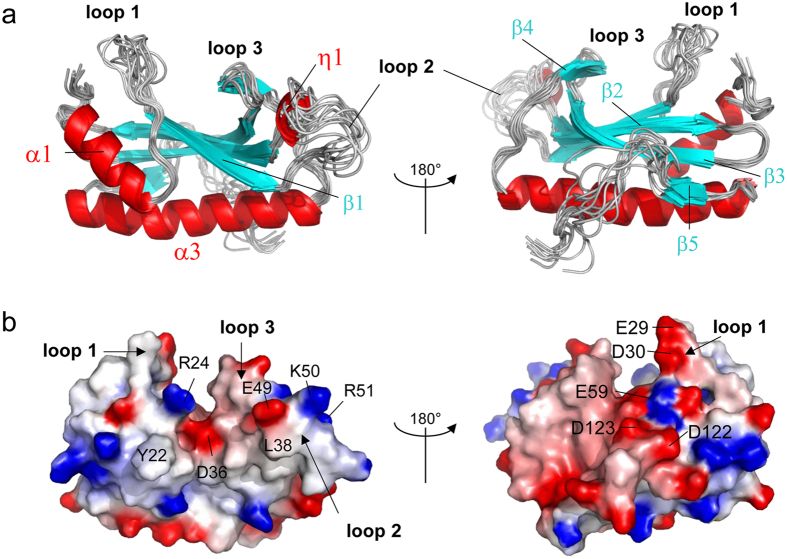
NMR structure of *S.* Typhimurium Crl. (**a**) Backbone ensemble structure of Crl_STM_ showing the 10 final low-energy conformers with the lowest CYANA target function in cartoon representation. Secondary structure elements are annotated using the numbering of Crl_PM_^18^. The two views are rotated by 180°. (**b**) The electrostatic surface potential was calculated with Delphi^44^ using the conformer 1. Colours are red to blue for acidic to basic potential. Critical residues, including D36, which delineate the entrance of the cavity, are indicated.

We previously reported the X-ray crystal structure of Crl from *Proteus Mirabilis* (Crl_PM_) (PDB 4Q11^18^), which suggested a high degree of flexibility of the protein. To get more insights into the dynamics of Crl, we solved the solution structure of Crl_STM_ by NMR^23^ (Fig. 5, Supplementary Table S2). Structural alignment with Crl_PM_ indicated that the fold of Crl_STM_ is conserved with a core consisting of a five-stranded β-sheet flanked by two helices, α1 and α3, with a cavity on top, closed by loops 1 and 2 (Supplementary Fig. S6). The electrostatic surface potential of Crl_STM_ delimits two faces of the protein, corresponding to lateral entries of the cavity (Fig. 5b). One face is overall neutral with several basic patches, whereas the opposite face is predominantly negatively charged, like loop 3 and the inside of the cavity, which are also rather acidic.

**Figure 6:**
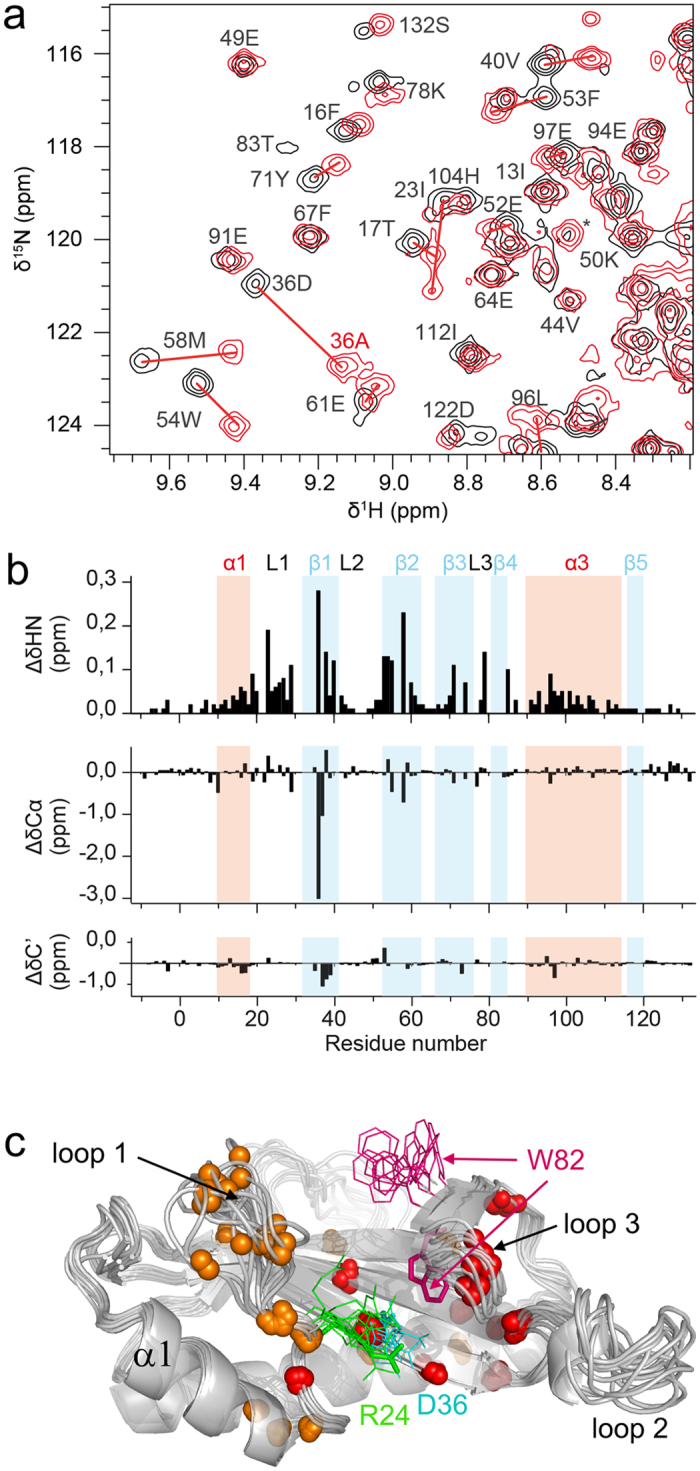
Structural analysis by NMR of the D36A Crl_STM_ mutant. (**a**) Selected region of assigned ^1^H-^15^N HSQC spectra of ^13^C^15^N-labeled D36A (red) and wild-type (black) Crl_STM_ displaying chemical shift perturbations due to the D36A substitution. Red lines link D36A to the corresponding wild-type Crl_STM_ signals. (**b**) Plot of combined ^1^H^15^N amide and ^13^Cα and ^13^C’ backbone CSPs as a function of residue numbers in Crl_STM_. Secondary structures are indicated by background coloring (red for α-helices and blue for β-strands) and annotated on top. Loops are denoted by the letter L. (**c**) Mapping of amide CSPs on a cartoon representation of an NMR ensemble structure (10 conformers) of Crl_STM_. Nitrogen atoms are shown by red and orange spheres for residues with ΔδHN > 0.1 ppm and >0.05 ppm, respectively. The side chains of R24, D36 and W82 are shown as lines in green, cyan and magenta, respectively. The corresponding side chains of a model built from the X-ray structure of Crl_PM_ (PDB 4Q11) are indicated in sticks with the same colours.

The NMR ensemble structure (Fig. 5a) showed that several regions at the periphery of the core display structural disorder. NMR signals in loop 1 (L19-F33) were broad, possibly due to conformational exchange at the millisecond timescale. Still a number of NOE contacts were found with α1 and β2, showing that it is not completely disordered. Due to the absence of the small helix α2 found in Crl_PM_, loop 1 of Crl_STM_ explores a wider space and contributes to forming a deeper cavity than in Crl_PM_ (Supplementary Fig. S6). Residues in loop 2 displayed sharp signals but only few NOE contacts, indicating that this region is disordered and flexible. The difference of dynamics in loop 2 as compared to the structured regions was also corroborated by ^15^N relaxation experiments, where it displays lower R_2_ rates that deviate from simulated R_2_ values (Supplementary Fig. S7). Finally the region corresponding to helix α4 in Crl_PM_ is not structured in Crl_STM_ (Supplementary Fig. S6b). Indeed, only few inter-residue NOE contacts were found in the P120-P128 region, but they provided evidence of the proximity between the C-terminus and helix α3. Strikingly, signals of several residues in the core β-sheet were broad, denoting conformational fluctuations that might be coupled to those in loop 1. The corresponding side chains could not be constrained during structure calculation, prominently that of W82 in strand β4, which points towards the cavity in the crystal structure, but appears to flip out in the NMR structures (Fig. 6c).

### Structural analysis of the Crl_STM_ D36A mutant

As shown previously, the Crl_STM_ D36A variant neither activates nor binds to σ^S^_STM_^18^, but it was not clear if this was due to structural alterations, since previous biophysical data suggested that the substitution could lead to partial loss of secondary and tertiary structure. Therefore we investigated the structural integrity of Crl_STM_ D36A by analysing its backbone chemical shifts. Signal overlap between wild-type and D36A Crl_STM_ allowed to partly transpose chemical shift assignments from wild type to D36A Crl_STM_ (Fig. 6a). But chemical shift perturbations (CSPs) were not restricted to the region of the mutation (Fig. 6b) and *de novo* backbone assignment had to be carried out. The data showed that there is no major difference for ^13^C’ or ^13^Cα chemical shifts, excepted for D36 and C37 (Fig. 6b), indicating that the secondary structure and overall fold are conserved in the mutant. In contrast, amide chemical shifts were significantly perturbed all over the sequence, even if the largest CSPs were also observed around the mutation. They seem to be relayed from D36 in strand β1 to β4, *via* β2 and β3, and to loops 1 and 3 (Fig. 6c). CSPs in loops 1 can be traced back to the salt bridge formed between the R24 guanidinium and the D36 carboxylate in the X-ray structure of Crl_PM_ as well as in most NMR conformers of Crl_STM_ (Fig. 6c). When Asp is replaced by Ala, this interaction is disrupted, allowing loop 1 more conformational freedom. Loop 3 could be affected by breaking the hydrogen bond between D36 and the W82 indole observed in the crystal structure of Crl_PM_ (Fig. 6c). This hydrogen bond is not present in the wild-type Crl_STM_ NMR structure, but it cannot be ruled out that it is transiently formed in solution. Amide CSPs inside the β-sheet, but far from position D36, could be explained by a slight reorganization of the hydrogen bond network. Altogether, these results endorse the role of residue D36 in σ^S^ binding.

### NMR analysis of the Crl_STM_ binding interface for σ^S^_STM_

**Figure 7:**
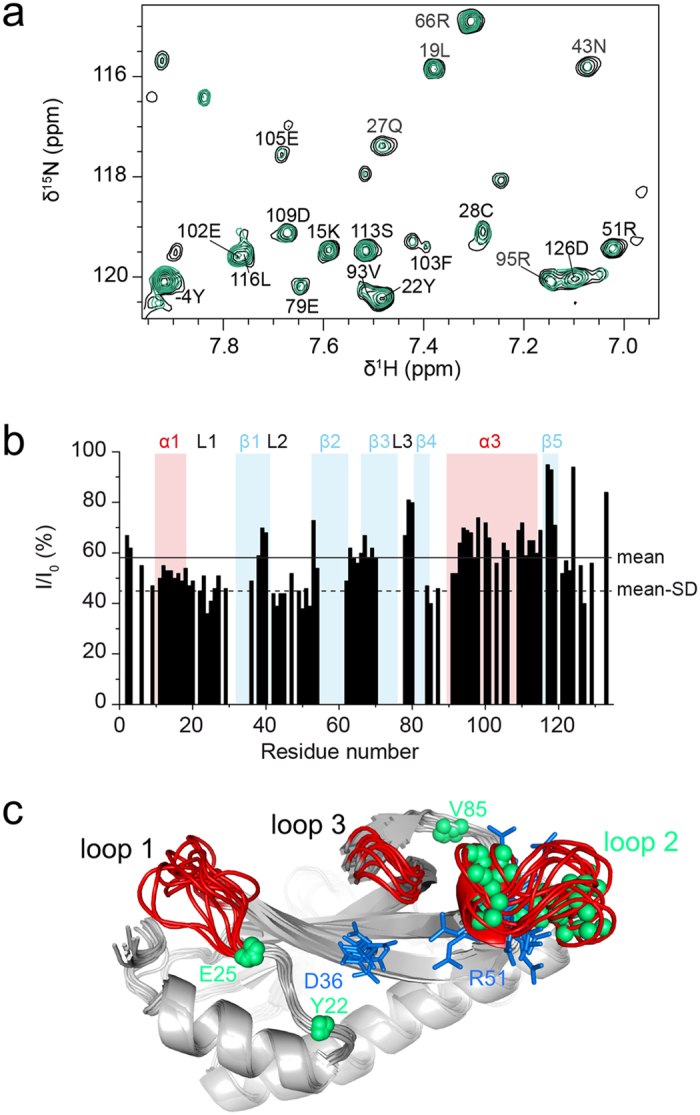
σ^S^_STM_-induced perturbations in NMR spectra of Crl_STM_. (**a**) Selected region of the ^1^H-^15^N TROSY spectrum of ^15^N^2^H-labeled Crl_STM_ in the absence (black) and presence of 0.25 equivalents of unlabelled σ^S^_STM_ (green) showing the intensity decrease of amide signals for some residues like N43. (**b**) Plot of intensity ratios as a function of residue number. Background colours indicate the boundaries of Crl_STM_ secondary structures (red for α-helices, blue for β-strands). The mean value and mean minus one standard deviation (SD) are shown in continuous and dashed lines. (**b**) The nitrogen atoms of residues with the lowest intensity ratios (I/I_0_ < mean—SD) in the presence of σ^S^ are shown in green spheres on the NMR structure of Crl_STM_, represented by three conformers to illustrate the structural variability in loop regions (in red). Two critical residues for σ^S^_STM_ binding, D36 and R51, are represented in blue sticks.

We next characterized the influence of σ^S^_STM_ on Crl_STM_ NMR spectra. ^1^H-^13^C HSQC spectra displayed line broadening, i.e. a decrease of intensities, in particular in the methyl region on addition of σ^S^ (Supplementary Fig. S8). Differential broadening was observed in loop 2 and helix α3. However, since methyl groups are mainly pointing to the inside of the structure and are not homogeneously distributed throughout the sequence, they may not be very sensitive probes for the Crl-σ^S^ interaction, which was suggested to rely on electrostatic interactions^18^. σ^S^_STM_ also induced overall line broadening in Crl_STM_^1^H-^15^N HSQC spectra, as a consequence of faster transverse relaxation in the Crl-σ^S^ complex than in free Crl, and additional line broadening for several residues (Fig. 7a,b, e.g. residue N43), due to exchange between free and complexed Crl. These are mainly clustered in loop 2 (Fig. 7b,c) which contains R51, one of the key residues for σ^S^ binding^17^,^18^. Since this region appears to be flexible in free Crl, the dynamics of loop 2 certainly plays a role in the formation of the Crl_STM_-σ^S^_STM_ complex. Helix α1 and loop 1 also seem to be affected by σ^S^_STM_ (Fig. 7b,c).

### Modeling of the σ^S^-Crl complex based on mutational analysis

Charged residues in Crl, D36 and R51, were previously found to be essential for σ^S^ binding^18^. The results above corroborate the hypothesis that the Crl-σ^S^ complex formation is likely driven by electrostatic interactions by demonstrating that one positively charged residue, R82 in σ^S^_STM_, is of paramount importance for Crl binding and activation. In addition, two acidic residues in σ^S^, D135 and E137, were spotted as likely candidates for interaction with Crl (Supplementary Fig. S1 and 21).

To integrate these data, we modelled the Crl-σ^S^ complex from a structural model of *S.* Typhimurium σ^S^_2_^16^ and the Crl_STM_ NMR structure. In a first step we performed normal mode analysis (NMA) on both Crl and σ^S^ to detect collective low-frequency motions that could provide conformations more favourable for complex formation than the starting structures of isolated proteins. In the case of σ^S^, although shearing movements take place between helix α2 and the DPE loop, residues R82 and E137 do not move wide apart and belong to a common interaction surface (Supplementary Fig. S9). In the case of Crl, collective motions of the three loops remodel the cavity either by closing it or widening it, which would help accommodating σ^S^_2_ (Supplementary Fig. S10).

In a second step, Crl-σ^S^ complexes were obtained *in silico*, using the information of critical binding residues and two different docking strategies. In the first strategy, we used the ZDock server^24^ in combination with refinement on the RosettaDock server^25^, that do not take into account conformational changes and flexibility of proteins (Models A to E, Supplementary Fig. S11). The second strategy used the Haddock Webserver^26^,^27^ to integrate the high degree of flexibility of the NMR structure of Crl (Supplementary Fig. S12).

In models A and B (Supplementary Fig. S11), σ^S^ R82 interacts with the Crl residues E25 or E102, respectively. These residues are not conserved in Crl family members^22^ and the Crl E25A and E102A variants interacted with σ^S^_STM_ in the same manner as wild type Crl (Supplementary Fig. S1) indicating that E25 and E102 are not required for σ^S^ binding. It is noteworthy that in these two models the DPE motif does not have any interacting partner. Altogether these data suggest that models A and B do not represent the Crl-σ^S^ interface. Models C and D are also unlikely since E137 in σ^S^ interacts with R24 in Crl, a residue dispensible for σ^S^ binding^18^. Furthermore, in model C, R82 in σ^S^ interacts with E25 in Crl, which is not involved in σ^S^ binding (Supplementary Fig. S1) and in both models R51 in Crl does not have any possible charged interacting partner in σ^S^.

**Figure 8:**
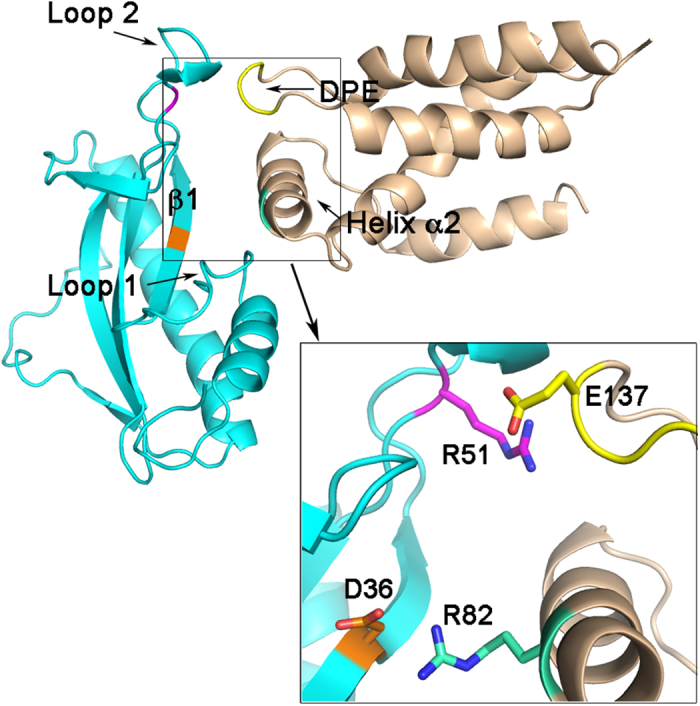
Cartoon representation of the proposed σ^S^-Crl interface. The most likely model (Model E, Supplementary Fig. S11) is shown with Crl depicted in cyan and σ^S^ in wheat. The region containing the two salt bridges between σ^S^ and Crl is zoomed. In the zoom view, the side chain of the charged residues, involved in salt bridges, are colored as follows: in Crl, R51 in orange, D36 in magenta and in σ^S^, R82 in green and the DPE motif in yellow.

From the five models generated by the first docking strategies, model E appears the more likely. In this model two salt bridges are formed involving the critical binding residues R82 and E137 in σ^S^ and D36 and R51 in Crl (Fig. 8). Moreover, several van der Waals and hydrogen bond interactions between the σ^S^ helix α2 and both loop 1 and β1 of Crl, and the σ^S^ loop containing the DPE motif and loop 2 of Crl, can further contribute to the σ^S^-Crl complex (Supplementary Fig. S13), in agreement with NMR data which suggest that also loop 1 in Crl is affected upon σ^S^_STM_ binding.

In the second series of docking experiments using Haddock Webserver^26^,^27^, pairs of active residues with complementary charges straightforwardly formed salt bridges (Supplementary Fig. S12), most often Crl-D36/σ^S^-R82. It was not possible to restrain the Crl-R51/σ^S^-E137 pair to form a salt bridge, but in a number of clusters the two loops that contain these two residues were in close contact, corroborating the relevance of model E for the σ^S^-Crl interface and in agreement with the NMR interaction experiments that pointed to the role of loop 2 for complex formation.

Residue D87 was previously pointed as important for Crl binding in *E. coli*^21^. The amino acid sequence of σ_2_ is identical in σ^S^_STM_ and σ^S^ from *E. coli* and, in the σ^S^_STM_ structural model, D87 is located at the edge of helix α2, with its side chain directed on the opposite face with respect to residue R82, as imposed by the geometry of an α-helix (Supplementary Fig. S14). Therefore D87 is unlikely to interact directly with Crl. Consistent with this hypothesis, in the study by Banta *et al.*^21^, some amino acid substitutions of D87, such as D87C, did not drastically affect the σ^S^ interaction with Crl.

## Discussion

In many Gram-negative bacteria, σ^S^/RpoS is the master regulator of gene expression in stress conditions and during stationary phase. σ^S^ is exquisitely and tightly regulated by many mechanisms that keep its production level and activity under strict control^3^,^4^,^5^. Crl is a unique regulatory factor, specifically dedicated to σ^S^, which enhances its activity, helping the association of σ^S^ with E^15^. Nevertheless, there are some *rpoS*-containing species, including *P. aeruginosa*, that do not harbour a *crl* gene^16^ and in which σ^S^ activity may be controlled by alternative mechanisms or functional homologs of Crl.

The strong sequence conservation of σ^S^_2_, the only σ^S^ domain that binds Crl^16^,^18^,^21^, prompted us to assess possible activation of σ^S^_PA_ by Crl. We show here that σ^S^_PA_ is not activated by Crl due to its inability to interact with Crl. Taking advantage of the evolution of the σ^S^ sequence in *P. aeruginosa* and other species lacking *crl*, we identified residues conserved in σ^S^ sequences from *crl* proficient species, and potentially implicated in Crl recognition. Among these, a surface-exposed arginine in σ^S^_STM_, R82, was assigned to the σ^S^-Crl interface. This residue is not conserved in σ^S^_PA_, which instead contains a leucine. Importantly, substitution of this leucine by an arginine rendered σ^S^_PA_ sensitive to Crl activation. It is noteworthy that, in some σ^S^ proteins from species that do not harbour *crl*, the arginine residue is conserved (Supplementary Fig. S2). It would be interesting to determine whether these σ^S^ proteins interact with Crl, and if not, whether they could be used to identify additional σ^S^ residues involved in Crl binding by the strategy described in this study for σ^S^_PA_.

The *in silico* models of the σ^S^-Crl complex show that salt bridges can indeed be formed for the two pairs of residues Crl-D36/σ^S^-R82 and Crl-R51/σ^S^-E137. In some models they can be formed simultaneously. This leads to a picture of an ideal binding interface in which helix α2 of σ^S^, containing R82, would dock into the cavity of Crl containing D36, disrupting the intermolecular R24-D36 contact, and the DPE motif and loop 2 of Crl would make contact on the outside, driven by electrostatic interactions between Crl-R51 and σ^S^-D135/E137 (Fig. 8).

What renders the σ^S^-Crl system very intriguing is its transitory and dynamic binding mechanism, which is unclear so far. Our NMR data together with the *in silico* modelling shed some light on how σ^S^ and Crl may interact and form a transient complex. The chemical shift perturbations in the NMR spectrum of Crl in the presence of σ^S^ indicate that loop 2 senses the presence of σ^S^, but extend beyond the region directly involved in σ^S^ binding, including helix α1, loop 1 and helix α3. These findings suggest that local structural rearrangements might take place in the flexible loops that allow breathing of the cavity as indicated by normal mode analysis of the Crl structure. Such rearrangements might contribute not only to the formation of the σ^S^-Crl complex, but also to its dissociation, once Crl has accomplished its work. Moreover, in free Crl, residue D36 is involved in an intramolecular interaction with R24. To form a new salt bridge with σ^S^-R82, the first one has to be broken. The perturbations observed in the NMR spectra of the Crl D36A variant show how the disruption of this network is sensed by the whole Crl structure, in particular by loop 1. It is tempting to speculate that this variant mimics the molecular processes that Crl undergoes upon σ^S^ binding, as we previously hypothesized^18^.

How does Crl binding to σ^S^ increase the σ^S^ association rate with E? Why is the σ^S^-Crl interaction so transient? These questions are still open. One possibility is that Crl triggers a conformational change in σ^S^ favouring its association with E. There is no high resolution 3D structure for free σ factors, but several biochemical and structural studies using the housekeeping σ^70^ have shown that σ factors undergo pronounced conformational changes upon E binding, allowing domains σ_2_ and σ_4_ to be spaced correctly for promoter binding^2^,^20^. These findings have led to the proposal that σ factors must be in a more compact conformation when free in the cell than in the Eσ complex. Consistent with this hypothesis, free σ are not able to bind promoters efficiently. This concept was further supported by the results obtained with engineered cysteine mutants of σ^28^, which showed that this σ factor has a compact conformation when free in solution^28^.

Modulation of the free σ^S^ conformation might be a common way to regulate both the stability and activity of σ^S^. σ^S^ is degraded by the ATP-dependent complex ClpXP protease^3^,^4^,^5^. However, σ^S^ binding by the RssB protein is required for delivery to ClpXP^3^,^4^,^5^. It has been postulated that RssB binding triggers a conformational opening of σ^S^ that exposes a ClpXP binding site, that is otherwise occluded in a closed conformation of free σ^S5^. Therefore, if the conformation of σ^S^ in the cell is rather compact, Crl binding to σ^S^_2_ may alleviate intramolecular interactions between σ^S^_2_ and other σ^S^ domains, favouring an open conformation for just the time required for σ^S^ to bind E, but transiently enough to avoid σ^S^ degradation by ClpXP. Further investigation of the structure of the σ^S^-Crl complex, for which a starting base is provided in the present study, and of the free σ^S^ conformation will assess the relevance of this scenario.

## Methods

### Bacterial strains, bacteriophage, plasmids and growth conditions

Strains and plasmids used for this work are listed in Supplementary Table S5. Bacteriophage P22HT105/1*int* was used to transfer mutations and the *katN-lacZ* fusion between *Salmonella* strains by transduction^29^. Green plates, for P22-infected cells or lysogens screening, were prepared as described previously^30^. Strains were grown in Luria-Bertani (LB) medium^31^ at 37°C under aeration. Development of the rdar morphotype was monitored on CR plates (LB agar without NaCl supplemented with Congo red 40 μg/ml and Coomassie brilliant blue R250 20 μg/ml), at 28°C as described^7^. Antibiotics were used at the following concentrations: ampicillin (Ap) 100 μg/mL; carbenicillin (Cb) 100 μg/mL; chloramphenicol (Cm) 15 μg/mL for the chromosomal resistance gene and 30 μg/mL for the plasmid resistance gene; kanamycin (Km) 50 μg/mL; and tetracycline (Tet) 20 μg/mL.

### *rpoS* allelic exchange in *Salmonella*

Allelic exchange of *rpoS* in *S.* Typhimurium ATCC14028 was achieved with a two-step Red-recombinase-based recombineering procedure^32^,^33^,^34^,^35^. The procedure involves replacement of the *tetRA* module of strain VFC326 by PCR-amplified DNA fragments of the *rpoS* allele from pVFC629, pVFD410, pVFD412 and pVFD399 (Supplementary Table S5 and S6) through positive selection of tetracycline-sensitive recombinants. All strains were confirmed to contain the expected mutation by DNA sequencing.

### Protein production and BACTH assays

The N-terminal (his)_6_-tagged σ^S^_PA_ wild type and variant L87R, σ^S^_STM_ R82L variant and Crl_STM_ were produced in *E. coli* strain BL21 (DE3) harbouring plasmid derivatives of pETM11 (Supplementary Table S5). Production and purification of the proteins were carried out as previously described^18^. ^15^N-, ^13^C^15^N- or ^15^N^2^H-labeled wild type (his)_6_- Crl_STM_ and ^15^N^13^C-labeled Crl_STM_ (his)_6_-D36A protein samples for NMR experiments were produced in minimum M9 medium^31^ supplemented with ^15^NH_4_Cl and unlabelled or ^13^C- or ^2^H-labeled glucose following the same protocol as^18^. Samples were subsequently dialyzed into NMR buffer (50 mM sodium or potassium phosphate, 300 mM NaCl or KCl, 2 mM dithiotreitol, at pH 8 or 7.5).

For bacterial adenylate cyclase-based two hybrid assay, the *E. coli cya* strain DHT1 was transformed with derivatives of plasmids pKT25 and pUT18 encoding σ^S^ and Crl proteins fused to the C-terminal part of T25 and the N-terminal part of T18, respectively (Supplementary Table S5). Co-transformants were plated onto MacConkey maltose plates supplemented with Cb, Km, and 0.5 mM IPTG to assess the Mal phenotype and on LB plates supplemented with X-Gal (40 μg/ml) Cb, Km, and IPTG (0.5 mM) to assess the Lac phenotype. Plates were incubated at 30 °C for 2 days and then isolated colonies were grown in LB supplemented with Cb, Km, and IPTG, at 30 °C for 20 hours. β-galactosidase activities were measured as described by Miller and are expressed in Miller units^36^.

### NMR experiments

NMR measurements were carried out at 293 K on a Bruker Avance III spectrometer with a magnetic field of 18.8 T (800 MHz ^1^H frequency) equipped with a cryogenic TCI probe. The magnetic field was locked with 7% or 100% ^2^H_2_O. Spectra were processed with Topspin 3.1 (Bruker Biospin) or NMRPipe^37^ and analysed with CCPNMR 2.2 software^38^. Chemical shift assignments of Crl_STM_ are reported elsewhere^23^. For structure determination, 2D ^1^H-^15^N HSQC and 3D ^1^H-^15^N NOESY-HSQC spectra were recorded from a 300 μM ^15^N-Crl sample, and ^1^H-^13^C HSQC, 2D ^1^H-^1^H NOESY and 3D ^1^H-^13^C NOESY-HSQC spectra from a 250 μM ^13^C^15^N-Crl sample in deuterium oxide buffer. NOESY mixing times were 80 ms.

Sequential backbone assignment of Crl_STM_ D36A (380 μM, 293 K) was carried out with a minimal set of triple resonance experiments: HNCA and HN(CO)CA were recorded at 14.1 T, CBCA(CO)NH and HNCO) at 18.8 T. Chemical shift perturbations induced by the D36A mutation were calculated as combined ^1^H and ^15^N perturbations ΔδHN for a given residue i:


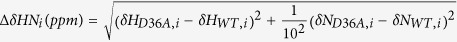


The scaling factor 1/10 corresponds to the gyromagnetic ratio difference between ^15^N and ^1^H.

### NMR structure calculation

NMR structures of wild-type Crl_STM_ were calculated using torsion angle dynamics in CYANA 2.2^39^. Backbone torsion angle restraints were generated with TALOS-N^40^ using Crl_STM_ backbone chemical shifts. Ambiguous distance restraints were collected from three sets of NOESY spectra and purged from 3D peaks without possible assignments in the ^1^H dimension bound to a heteroatom. The disordered N-terminal His-tag (His(−20)-His(0)) was excluded from structure calculation. Structure statistics were obtained from the Protein Structure Validation Server, version 1.5 (http://psvs-1_5-dev.nesg.org/) (Supplementary Table S2). Normal Mode Analysis was performed on single conformers on the ElNémo webserver^41^.

### Protein-protein docking

Rigid-body docking was carried out first on the ZDock server^24^, which employs a fast Fourier transform (FFT) algorithm, to generate the initial models (about 100) for the σ^S^-Crl complex with Crl_STM_ as the receptor and a homology model of *S.* Typhimurium σ^S^_2_ as the ligand^16^. Five models selected from ZDock were further refined using RosettaDock^25^, which performs a searching for the lowest-energy binding interface structures giving as ouput the 10 best-scoring models from 1000 total models. The presence of a ‘docking funnel’ was verified, considering that at least three of the first five lowest-energy binding interface models have a value of I_rmsd < 4 Å^42^ (Supplementary Table S3).

Flexible docking was carried out on the guru interface of the Haddock Webserver^26^,^27^ using single conformers from the NMR structure ensemble of Crl_STM_ and the homology model of *S.* Typhimurium σ^S^_2_. D36 and R51 in Crl and R82, D135 and E137 in σ^S^ were defined as active residues. Passive residues were automatically defined around active residues. Loops 1 (E25-R32) and 2 (N43-E52) in Crl and the DPE loop (K133-F142) in σ^S^ were defined as fully flexible. 1000 initial structures were generated. 200 final structures were refined in water and clustered according to RMSD criterion. Statistics for clusters obtained for the conformers 1 and 2 are given in Supplementary Table S4.

### Illustrations

Visualization and graphic rendering of protein structures were prepared with PyMOL^43^.

### Other methods

Methods for DNA manipulation, immunoblot analysis of proteins and CD and ITC experiments are described in Supplementary Methods.

## Additional Information

**How to cite this article**: Cavaliere, P. *et al.* Binding interface between the Salmonella σ^S^/RpoS subunit of RNA polymerase and Crl: hints from bacterial species lacking *crl*. *Sci. Rep.* **5**, 13564; doi: 10.1038/srep13564 (2015).

## Supplementary Material

Supplementary Information
