# Supplementary material for: Binding interface between the Salmonella σS/RpoS subunit of RNA polymerase and Crl: hints from bacterial species lacking crl
Source: Sci Rep. 2015 Sep 4;5:13564. doi: 10.1038/srep13564 (PMC4559669; doi:10.1038/srep13564)
Supplement: Supplementary Information [file srep13564-s1.pdf]

SUPPLEMENTARY DATA

Binding interface between the *Salmonella* σ^S/RpoS subunit of RNA polymerase and Crl: hints from bacterial species lacking *crl*

Paola Cavaliere[1,2], Christina Sizun[3], Fabienne Levi-Acobas[4,5], Mireille Nowakowski[4,5], Véronique Monteil[1,2], François Bontems[3], Jacques Bellalou[4,5] Claudine Mayer[5,6,7] and Françoise Norel[1,2]*

[1] Institut Pasteur, Laboratoire Systèmes Macromoléculaires et Signalisation, Département de Microbiologie, 25 rue du Docteur Roux, 75015 Paris, France

[2] CNRS ERL3526, rue du Docteur Roux, 75015 Paris, France

[3] Institut de Chimie des Substances Naturelles, CNRS UPR2301, 91190 Gif-sur-Yvette, France

[4] Institut Pasteur, Plate-forme de Protéines Recombinantes, Département de Biologie Structurale et Chimie, 25 rue du Docteur Roux, 75015 Paris, France

[5] CNRS UMR 3528, rue du Dr. Roux, 75015 Paris, France

[6] Institut Pasteur, Unité de Microbiologie Structurale, Département de Biologie Structurale et Chimie, 25 rue du Docteur Roux, 75015 Paris, France

[7] Université Paris Diderot, Sorbonne Paris Cité, Paris, France.

[*] Corresponding author : Email: francoise.norel@pasteur.fr Tel: (33) 140613122; Fax: (33) 145688960

Supplementary Figures and Legends

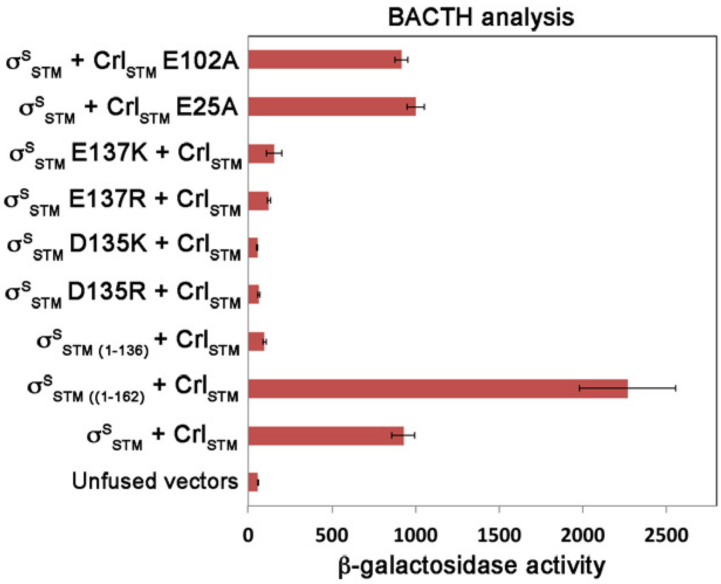

Supplementary Figure S1. BACTH interaction between *S.* Typhimurium Crl and σ^S proteins. (a) Interactions between the indicated T25-σ^S and Crl-T18 fusion proteins were quantified by measuring β-galactosidase activity in Miller units. Results are the mean of at least three independent experiments and standard deviations are indicated with black bars.

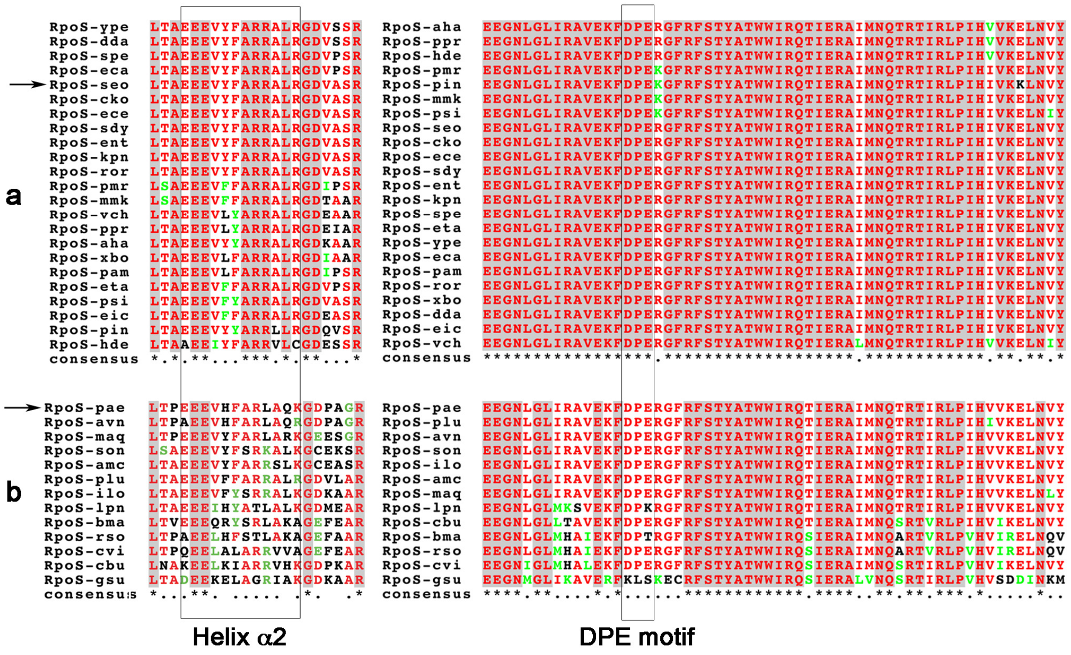

Supplementary Figure S2. Sequence alignment of σ^S/RpoS regions containing the helix α2 and the DPE motif from species harbouring crl (a) and species lacking crl (b). The σ^S sequences used are from the KEGG server (http://www.genome.jp/kegg/kegg2.html) with the following organism code: ype, *Yersinia pestis*; dda, *Dickeya dadantii*; spe, *Serratia protea maculans*; eca, *Pectobacterium atroseptium*; seo, *Salmonella* enterica serovar Typhimurium; cko, *citrobacter koseri*; ece, *Escherichia coli* (EHEC); sdy, *Shigella dysenteriae*; ent, *Enterobacter* sp. 638; kpn, *Klebsiella pneumoniae*; ror, *Raoultella ornithinolytica*; pmr, *Proteus mirabilis*; mmk, *Morganella morganii*; vch, *Vibrio cholera*; ppr, *Photobacterium profundum*; aha, *Aeromonas hydrophila*; xbo, *Xenorhabdus bovienii*; pam, *Pantoea ananatis*; eta, *Erwinia tasmaniensis*; psi, *Providencia stuartii*; eic, *Edwardsiella ictaluri*; pin, *Psychromonas ingrahamii*; hde, *Candidatus hamiltonella defensa* (In Panel a); pae, *Pseudomonas aeruginosa*; avn, *Azotobacter vinelandii*; maq, *Marinobacter hydrocarbonoclasticus*; son, *Shewanella oneidensis*; amc, *Alteromonas macleodii*; plu, *Photorhabdus luminescens*; ilo, *Idiomarina Ioihiensis*; lpn, *Legionella pneumophila*; bma, *Burkholderia mallei*; rso, *Ralstonia solanacearum*; cvi, *Chromobacterium violaceum*; cbu, *Coxiella burnetii*; gsu, *Geobacter sulfurreducens* PCA (In Panel b). The helix α2 and the DPE motif (corresponding to residues 74-85 and 135-137 in σ$^S_{STM}$, respectively) are highlighted. The alignments were generated with ClustalW[44]. The arrows indicate the sequences of σ$^S_{STM}$ and σ$^S_{PA}$ proteins characterized in this study.

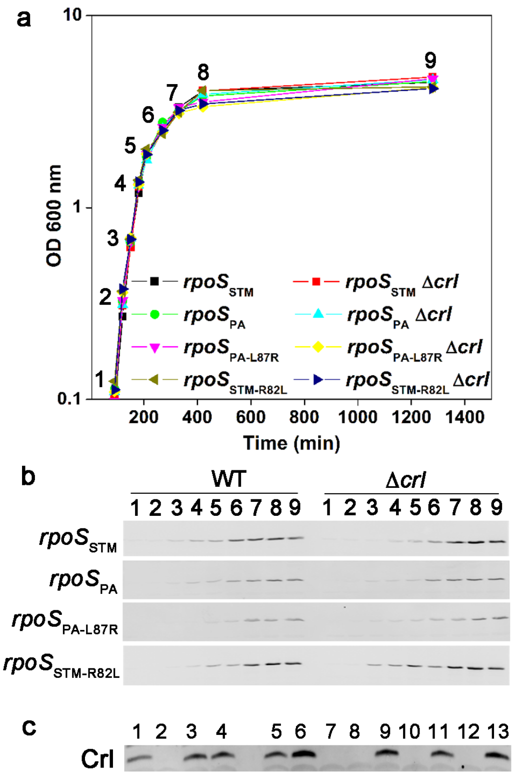

Supplementary Figure S3. Expression of σ^S and Crl in *S.* Typhimurium strains used in this study. (a) Growth of *Salmonella* strains containing the *katN-lacZ* fusion and harbouring the *rpoS* alleles indicated, and their Δ*crl* derivatives, was determined by measuring the culture OD_{600}. Aliquots were taken at different times points (time points 1 to 9) and assayed for β−galactosidase activity (Figure 3) and (b) analyzed by immunoblotting with antibody directed against σ^S_{STM}. 10 µg of total proteins was loaded in each slot. (c) Immunodetection of Crl, using antibody directed against Crl_{STM}, in overnight cultures of the following strains: lane 1, *S*. Typhimurium ATCC14028; lane 2, *Pseudomonas aeruginosa* VFC278; lane 3, ATCC14028 *rpoS*$_{PA}$, lane 4, ATCC14028 *rpoS*$_{PA-L87R}$; lane 5, ATCC14028; lane 6, ATCC14028 *rpoS*$_{STM-R82L}$; lane 7, ATCC14028 Δ*crl*; lane 8, ATCC14028 *rpoS*$_{STM-R82L}$ Δ*crl*; lane 9, ATCC14028 *rpoS*$_{PA}$; lane 10, ATCC14028 *rpoS*$_{PA}$ Δ*crl*; lane 11, ATCC14028 *rpoS*$_{PA-L87R}$; lane 12, ATCC14028 *rpoS*$_{PA-L87R}$ Δ*crl*; lane 13, ATCC14028 Δ*rpoS*. Experiments were repeated twice with similar results.

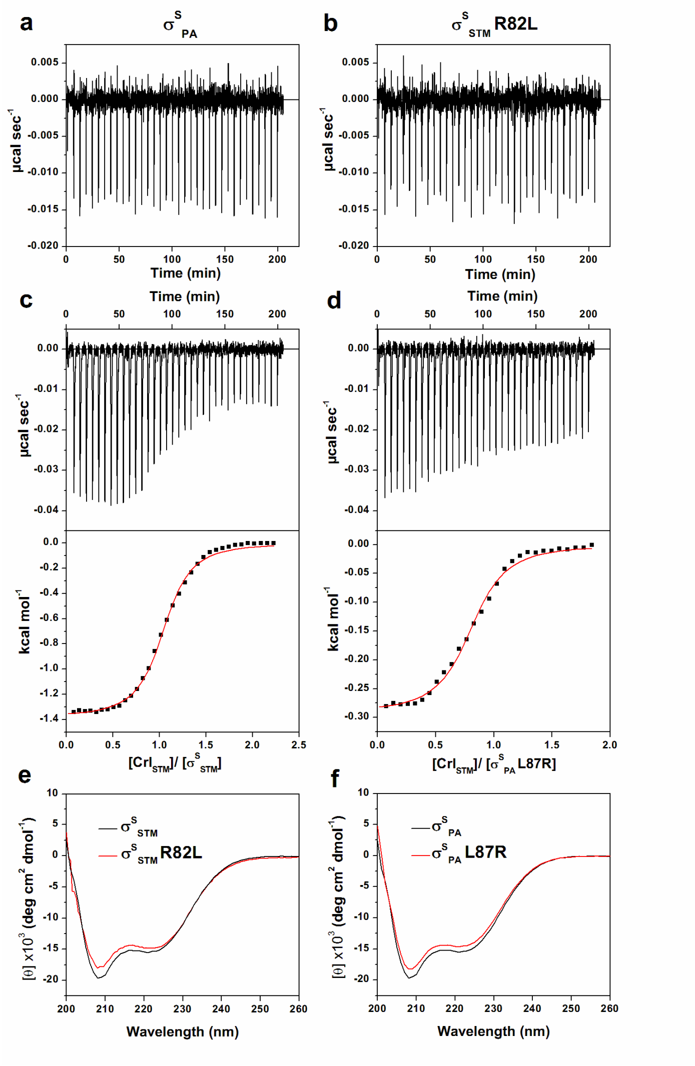

Supplementary Figure S4. ITC binding experiments between $\mathrm{Crl_{STM}}$ and σ^S proteins. Raw profiles are shown for ITC assays performed between $\mathrm{Crl_{STM}}$ and wild type $\sigma^S{}_{PA}$ (a) or $\sigma^S{}_{STM}$ R82L (b). ITC assays performed between $\mathrm{Crl_{STM}}$ and wild type $\sigma^S{}_{STM}$ (c) or $\sigma^S{}_{PA}$ L87R (d). ITC raw profiles (top of panels c and d) and integrated heat values plotted as a function of σ^S-Crl molar ratio (bottom of panels c and d) are shown. Far-UV CD spectra recorded in the range 200-260 nm for $\sigma^S{}_{STM}$, $\sigma^S{}_{STM}$ R82L (e), $\sigma^S{}_{PA}$ and $\sigma^S{}_{PA}$ L87R (d).

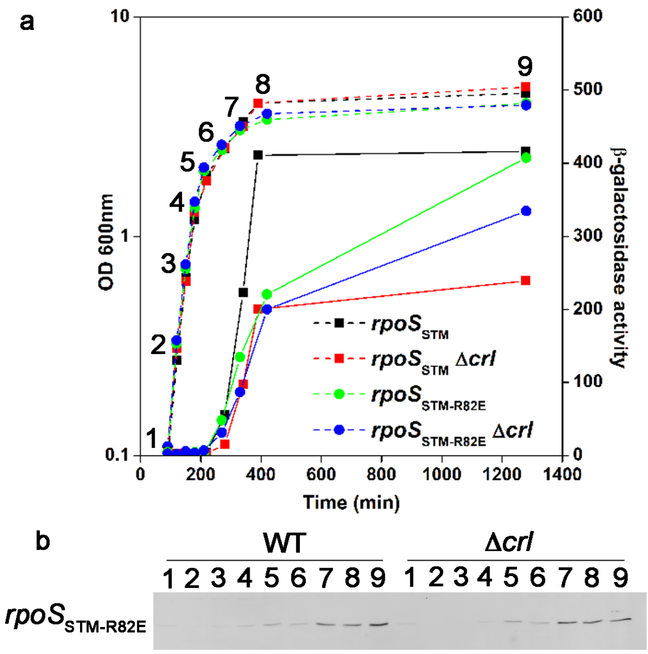

Supplementary Figure S5. Kinetic of expression of *katN-lacZ* and σ^S_{STM} in *S.* Typhimurium carrying the *rpoS*$_{STM-R82E}$ allele. (a) Growth (dashed lines) of *Salmonella* strains harbouring the *rpoS* alleles indicated and their Δ*crl* derivatives was measured by the culture OD_{600}. Aliquots were taken at different times points (time points 1 to 9) and assayed for β-galactosidase activity (solid lines). The experiments were repeated twice and a representative experiment is shown. (b) Immunodetection of σ^S_{STM} R82E in ATCC14028 *rpoS*$_{STM-R82E}$ and ATCC14028 *rpoS*$_{STM-R82E}$ Δ*crl* strains at time points 1 to 9 during growth (panel a), using antibody directed against σ^S_{STM}.

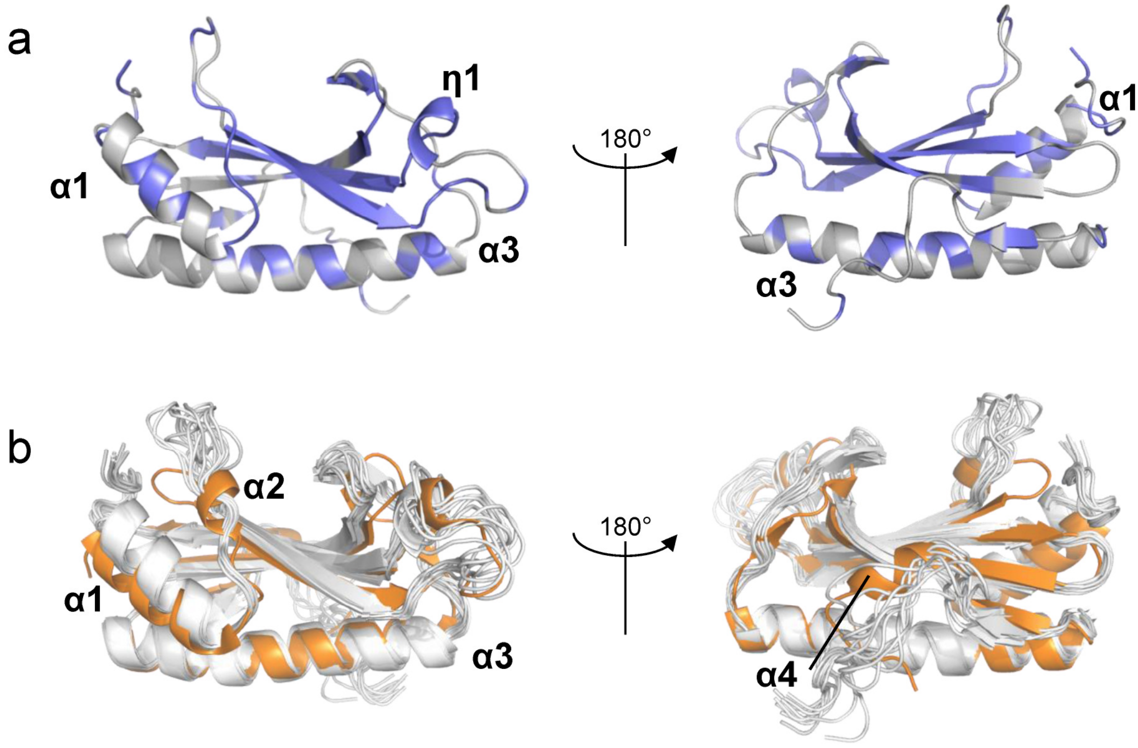

Supplementary Figure S6. Comparison between the Crl_{STM} NMR and Crl_{PM} X-ray structures. (a) Identical residues in the sequences of Crl_{PM} and Crl_{STM} are shown in blue on the first conformer of the NMR ensemble structure in gray cartoon. The two views are rotated by 180°. (b) The NMR ensemble structure (10 conformers, in white) was structurally aligned with the X-ray crystal structure of CrlPM (PDB 4Q11, chain A, in orange). Helix α2 is absent from the NMR structure of Crl_{STM} and α4 appears to be disordered.

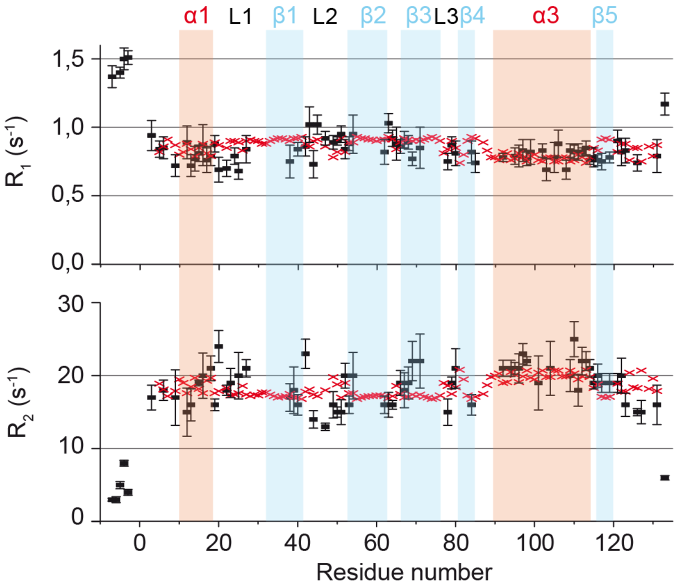

Supplementary Figure S7. ^{15}N relaxation measurements of $\mathbf{Crl_{STM}}$.. ^{15}N relaxation R_1 and R_2 relaxation rates were measured with ^{2}H,^{15}N labeled WT Crl by using TROSY transfers and a pseudo3D version recorded in an interleaved fashion with a recycling delay of 4.5 s. Relaxation delays were 10, 100, 200, 400, 600, 800, 1000, 1500 ms for R_1 measurements and 17, 34(*2), 68, 102, 136, 271 ms for R_2. Peak intensities were extracted in CCPNMR and relaxation curves were fitted to a monoexponential model. Error bars are standard deviations produced by covariance matrix analysis in CCPNMR[38]. Red symbols correspond to ^{15}N R_1 and R_2 nuclear relaxation rates simulated with HYDRONMR[45].

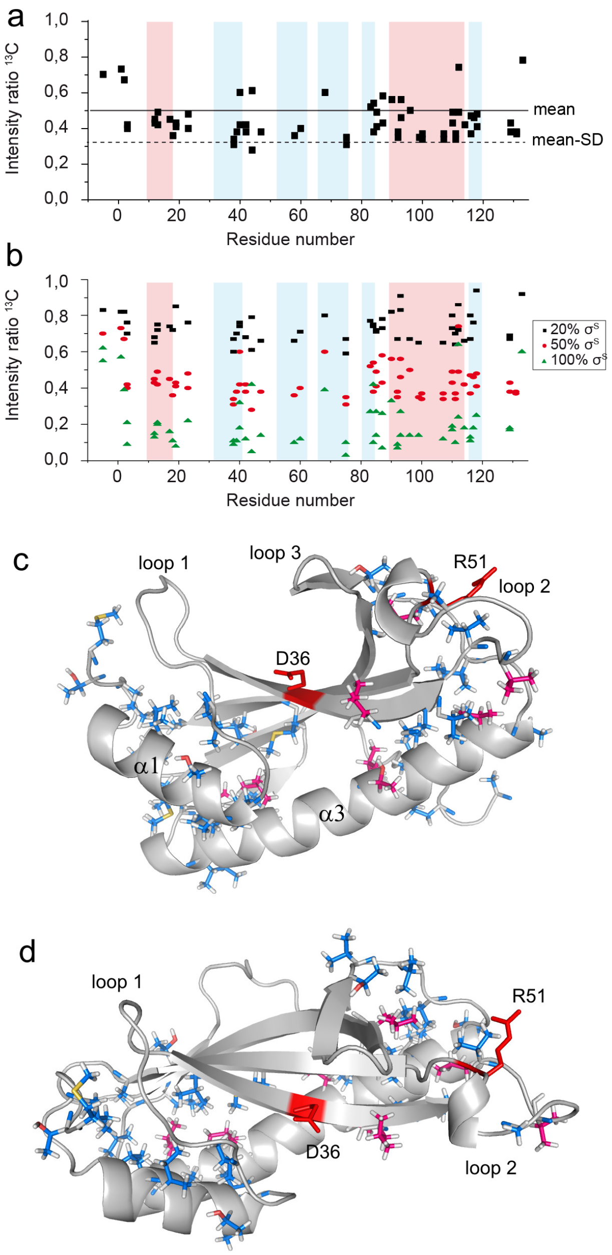

Supplementary Figure S8. Mapping of σ^S_{STM}-induced intensity perturbations in ^{1}H-^{13}C HSQC spectra of Crl_{STM}. (a) and (b) Intensities of methyl signals were measured from ^{1}H-^{13}C HSQC spectra in the presence of 0.5 molar equivalent of σ^S_{STM} (a) and with increasing amounts of σ^S_{STM} (b). Intensity ratios with respect to the reference spectrum without σ^S were plotted as a function of the residue number. (c) and (d) The residues with the lowest ratios, i.e. largest line-broadening, were mapped on the first conformer of the Crl_{STM} NMR structure, in magenta sticks according to a cut-off value corresponding to the mean value minus one standard deviation (indicated with broken lines in (a)), while all other residues with methyl groups are in marine. The critical residues D36 and R51 are represented with red sticks. The two views are rotated by 90°.

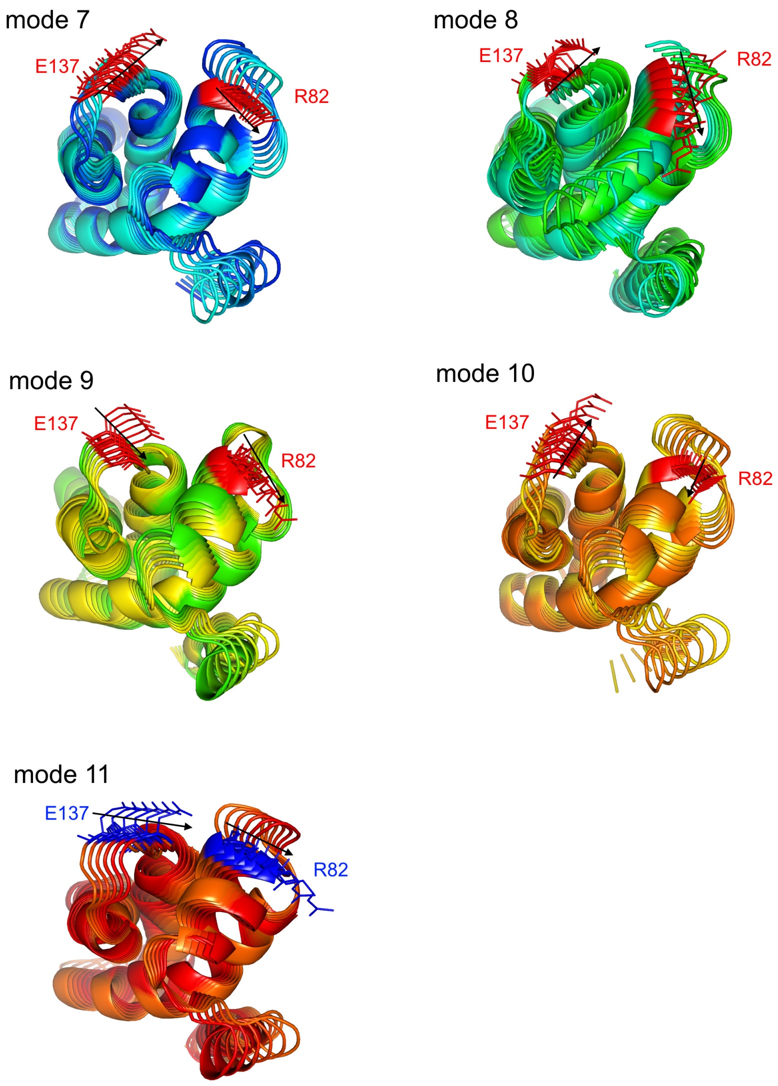

Supplementary Figure S9. Normal Mode Analysis of σ^{S}_{STM} structure. Normal mode analysis of the homology model[16] of σ^{S}_{STM} domain 2 was carried out on the ElNémo server using the Elastic Network Model[41]. Five low frequency normal modes are shown. The relative movements of helix α2 and the DPE loop are indicated by arrows. The critical residues R82 and E137 are shown in sticks, either in red or blue to contrast with the cartoon color.

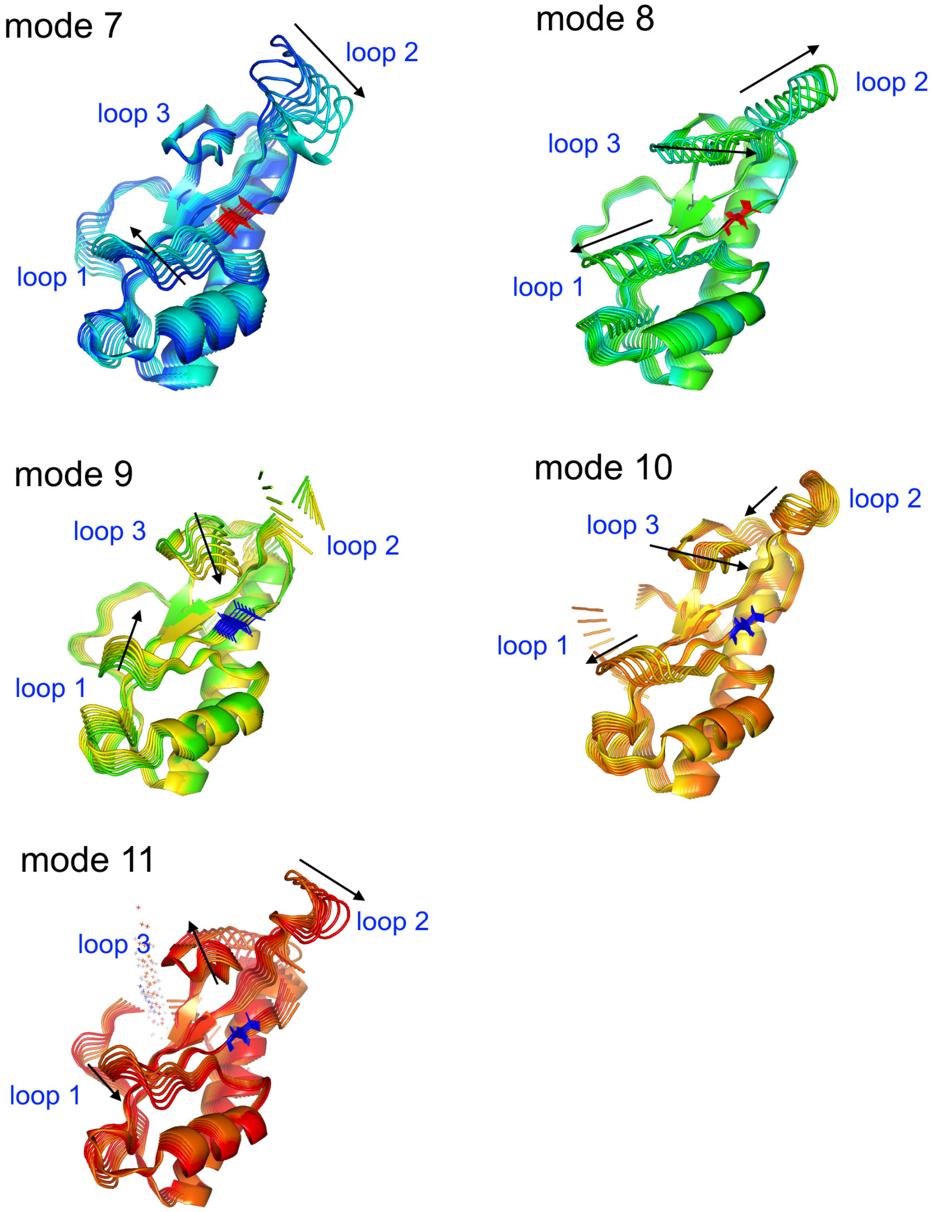

Supplementary Figure S10. Normal Mode Analysis of Crl_{STM} structure. Normal mode analysis of Crl_{STM} (conformer 2 of the NMR structure ensemble) was carried out on the ElNémo server using the Elastic Network Model[41]. Five low frequency normal modes are shown. They display large movements of the three loops indicated by arrows. Six structure models are shown in cartoon representation for each mode, including the starting structure and the structure with largest amplitude. Residue D36 is shown in sticks, either in red or blue to contrast with the cartoon color. Modes 7, 8, 10 and 11 could enhance access to the cavity by a shearing movement of loops 1 and 2, loops 1, 2 and 3, loops 1 and 3, and loops 2 and 3 respectively. Mode 9 closes the cavity by bending loops 1 and 3 towards each other.

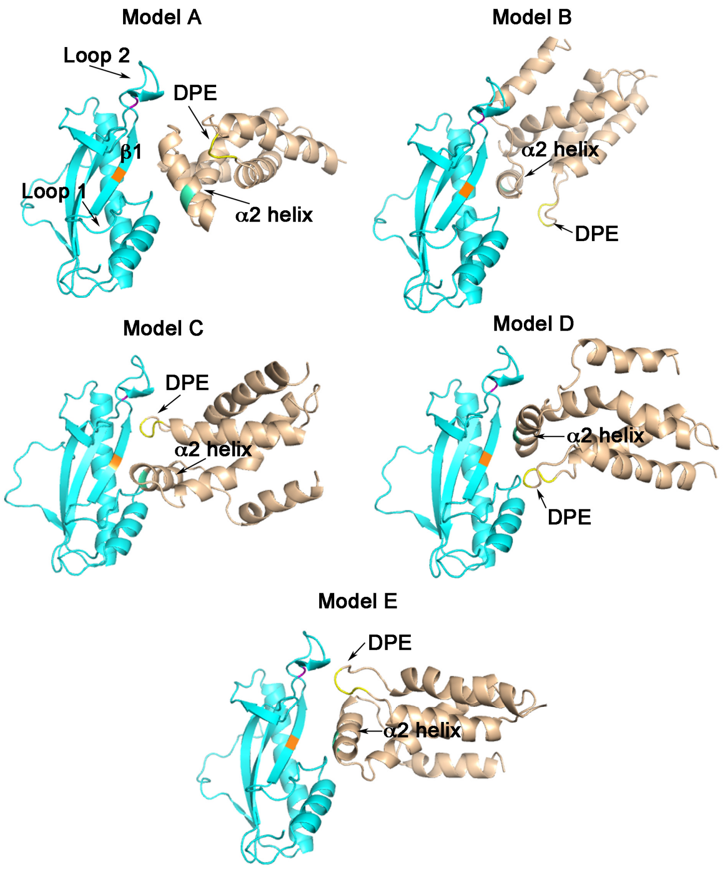

Supplementary Figure S11. σ^S-Crl complex models obtained by RosettaDock, using the structural model of *S.* Typhimurium σ^S_2 and the Crl_{STM} NMR structure. Cartoon representation of the five models refined by RosettaDock[42]. Crl_{STM}, shown in cyan, is kept in the same orientation in all models and σ^S_{STM}, shown in wheat, has different orientations depending on the model. For Crl_{STM} and σ^S_{STM} residues, the same color code as in Figure 8 was used.

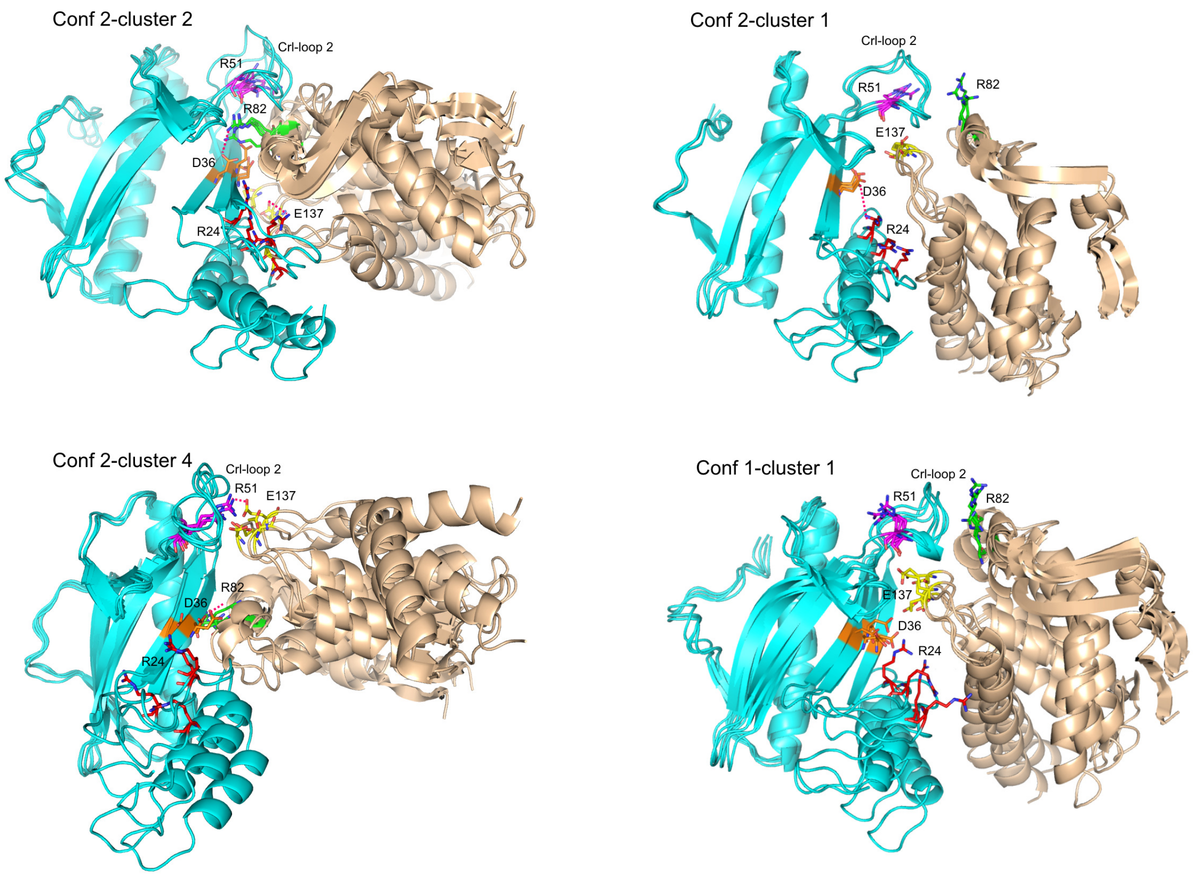

Supplementary Figure S12. σ^{S}_{2}-Crl$_{STM}$ complex models obtained using the Haddock Webserver[26,27] with flexible loops in both σ^{S} and Crl. The complexes were generated as described in Materials and Methods with Crl-D36 and -R51 and σ^{S}-R82, -D135 and -E137 as active residues, and Crl loops 1 and 2 and σ^{S}-DPE loop declared as fully flexible. The flexibility of loop 1 combined to the absence of constraints on Crl helix α1 induces a large displacement of helix α1 in several complex model. β-strand 1 can also be slightly displaced. Top clusters (according to the HADDOCK score, see Supplementary table S4) obtained with the Crl conformers 1 or 2 of the NMR ensemble structure are represented by four structural models each. Crl$_{STM}$, shown in cyan, is kept approximately in the same orientation in all models. σ^{S}_{STM} is shown in wheat. For the five critical Crl$_{STM}$ and σ^{S}_{STM} residues, the same color code as in Fig. 8 was used. The four clusters display different orientations for σ^{S}_{STM} depending on the pairs formed by the five residues (indicated by magenta broken lines). In the cluster "Conf 2-cluster4", a salt bridge is formed between σ^{S}_{STM}-R82 and Crl-D36, and residues σ^{S}_{STM}-E137 and Crl-R51 are close to each other, which is reminiscent of model E generated by Z-Dock[24] and refined by RosettaDock[25] (Supplementary Fig. S11).

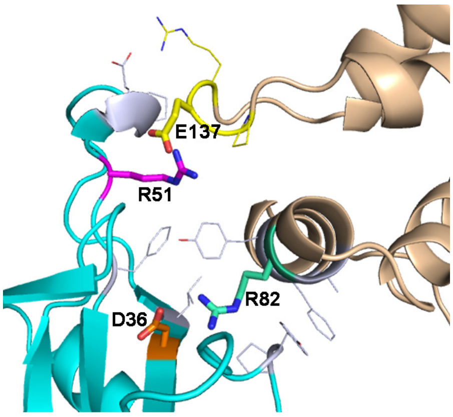

Supplementary Figure S13. σ^S-Crl binding interface of Model E. Van der Waals and hydrogen bond interactions are established between several residues within σ^S helix α2 and both loop 1 and β1 of Crl, and the σ^S loop containing the DPE motif and loop 2 of Crl. The residues involved in these interactions are shown as lines in grey in both σ^S and Crl proteins, whilst R82 and the DPE motif in σ^S and D36 and R51 in Crl are shown as sticks with the same color code as in Figure 8.

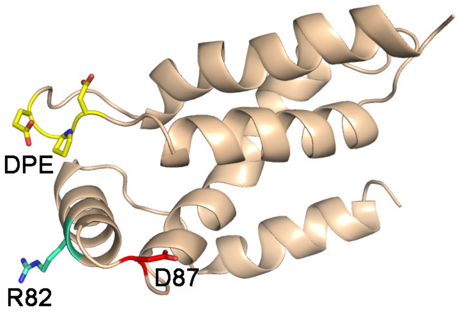

Supplementary Figure S14. Orientation of the side chain of residue D87 in the structural model of σ^S_{STM}. The side chain of D87 is directed on the opposite face with respect to residue R82.

Supplementary Tables

Supplementary Table S1. Thermodynamic parameters of the interaction of σ^S_{STM} and σ^S_{PA} L87R with Crl_{STM} obtained by ITC at 25°C

Each ITC experiment was performed in triplicate and standard deviations are reported.

	N	K_D (μM)	Δ_bH (kJ/mol)	$T\Delta_bS$ (kJ/mol)	Δ_bG (kJ/mol)
σ^S_{STM} + Crl_{STM} [18]	1.0 ± 0.1	0.8 ± 0.2	-8.2 ± 0.5	27 ± 1	-35 ± 1
σ^S_{PA} L87R + Crl_{STM}	0.88 ± 0.1	1.2 ± 0.5	-1.5 ± 0.6	30 ± 1	-31 ± 1

Supplementary Table S2. NMR restraints and structural statistics[a] for *S.* Typhimurium Crl

Number of structures	20
Number of NOE distance constraints	
Total	1607
Intra-residue (i = j)	437
Sequential |i - j| = 1	405
Medium range: 1 <|i - j| <5	238
Long range: |i - j| ≥ 5	527
NOE constraints per restrained residue	12.1
Number of dihedral angle constraints	105Φ, 112Ψ
Violation statistics[b]	
Distance violation/structure	
0.1 - 0.2 Å	2.05
0.2 - 0.5Å	0.45
> 0.5 Å	0
RMS of distance violation / constraint	0.01 Å
Maximum distance violation	0.38 Å
Dihedral angle violation/structure	
1-10°	14.1
>10°	0
RMS of dihedral angle violation / constraint	0.59°
Maximum dihedral angle violation	4.80°
Rmsd from ideal covalent geometry	
Bonds	0.001 Å
Angles	0.2°
Rmsd to mean coordinates	
Backbone	1.1/1.0 Å (all/ordered)
Heavy atoms	1.7/1.5 Å (all/ordered)
Ramachandran plot[c]	
Most favoured	89.8 %
Additionally allowed	10.2 %
Generously allowed	0.0 %
Disallowed	0.0 %

[a]Calculated using PSVS version 1.5 (http://psvs-1_5-dev.nesg.org/).
[b]Average distance constraints were calculated using the sum of r^{-6}.
[c]Calculated with Procheck[46].

Supplementary Table S3. Final score data obtained from RosettaDock server of the five initial ZDock models submitted. The I_rmsd values were checked to verify which models reached a docking funnel and are therefore relevant as σ^S_{STM}-Crl_{STM} binding interface.

	Total score[a]	I_rmsd[b]
Model A		
1	-173.62	9.951
2	-173.605	9.725
3	-173.43	5.077
4	-173.2	6.559
5	-173.135	3.364
Model B		
1	-173.059	7.508
2	-172.737	8.163
3	-172.315	6.066
4	-172.051	7.018
5	-171.857	7.566
Model C		
1	-171.735	2.061
2	-171.598	2.075
3	-170.178	1.461
4	-170.113	1.777
5	-169.821	1.828
Model D		
1	-172.214	9.625
2	-171.771	7.18
3	-171.744	9.364
4	-171.664	6.414
5	-171.641	9.122
Model E		
1	-173.536	3.608
2	-172.899	3.694
3	-172.721	3.553
4	-172.694	3.59
5	-172.374	3.776

[a]Total score : Total energy of the output interface with respect to the input model

[b]I_rmsd : Interface backbone root mean squared deviation

Supplementary table S4. Statistics for Crl-σ^S docking and clustering with Haddock Webserver.

Cluster	Haddock score [a.u]	Cluster size	RMSD [Å]	Van der Waals energy [kcal/mol]	Electrostatic energy [kcal/mol]	Desolvation energy [kcal/mol]	Restraints violation energy [kcal/mol]	Buried Surface Area [$Å^2$]	Z-Score
**Conformer 1**									
**4**	-101.8 ± 8.8	17	18.0 ± 0.8	-28.3 ± 8.2	-278.7 ± 106.6	-17.8 ± 8.6	0.0 ± 0.0	1183 ± 95	-2,0
**1**	-96.8 ± 6.0	27	8.7 ± 0.6	-32.8 ± 12.1	-155.4 ± 75.7	-35.4 ± 9.1	24.7 ± 10.0	1294 ± 149	-1,4
**7**	-86.6 ± 13.6	10	16.1 ± 0.5	-36.1 ± 9.1	-163.5 ± 43.7	-19.4 ± 7.8	15.4 ± 14.0	1323 ± 214	-0,2
**9**	-84.8 ± 18.0	9	3.3 ± 1.9	-26.4 ± 12.3	-195.2 ± 24.3	-20.1 ± 8.0	7.8 ± 13.5	1121 ± 335	0,0
**13**	-82.9 ± 10.5	4	14.6 ± 0.9	-26.5 ± 8.2	-141.4 ± 21.6	-28.1 ± 4.4	0.3 ± 0.5	1116 ± 202	0,2
**2**	-82.7 ± 3.9	26	8.1 ± 2.4	-24.8 ± 4.7	-151.1 ± 66.2	-28.5 ± 8.5	8.8 ± 15.0	929 ± 54	0,2
**8**	-82.6 ± 12.2	10	15.3 ± 0.5	-21.4 ± 5.0	-205.2 ± 45.7	-21.3 ± 8.8	10.6 ± 11.8	987 ± 177	0,2
**11**	-80.7 ± 7.1	6	14.1 ± 0.6	-27.2 ± 8.2	-111.0 ± 64.4	-31.7 ± 6.7	4.0 ± 4.3	1050 ± 150	0,5
**3**	-76.0 ± 4.4	23	14.0 ± 0.3	-23.2 ± 6.4	-114.2 ± 69.8	-30.1 ± 10.6	1.8 ± 2.2	930 ± 116	1,0
**10**	-71.0 ± 16.9	9	12.2 ± 0.8	-26.9 ± 8.9	-131.1 ± 62.4	-18.5 ± 2.3	6.8 ± 6.43	974 ± 346	1,6
**Conformer 2**									
**2**	-137.8 ± 11.9	38	2.8 ± 1.6	-46.5 ± 6.7	-232.8 ± 66.5	-46.5 ± 6.1	17.7 ± 17.8	1566 ± 123	-1,8
**1**	-125.4 ± 3.7	59	9.5 ± 0.5	-47.1 ± 6.3	-186.2 ± 33.6	-41.2 ± 9.5	1.3 ± 1.1	1609 ± 159	-1,3
**4**	-105.7 ± 9.1	20	16.4 ± 0.2	-24.2 ± 3.9	-235.3 ± 54.7	-34.4 ± 11.0	0.5 ± 0.5	1155 ± 158	-0,4
**6**	-99.4 ± 16.4	6	14.7 ± 0.8	-28.1 ± 11.1	-161.8 ± 107.5	-39.2 ± 9.6	2.1 ± 0.8	1124 ± 255	-0,1
**3**	-99.4 ± 4.6	31	14.5 ± 0.5	-34.3 ± 4.7	-122.8 ± 13.2	-41.3 ± 5.7	8.8 ± 14.0	1134 ± 52	-0,1
**7**	-81.7 ± 8.8	4	14.9 ± 0.6	-23.5 ± 4.6	-191.1 ± 69.9	-20.0 ± 18.4	0.0 ± 0.0	971 ± 109	0,7
**8**	-80.8 ± 27.3	4	4.0 ± 0.5	-28.8 ± 16.0	-158.0 ± 43.0	-21.3 ± 4.0	8.9 ± 14.8	1079 ± 443	0,7
**5**	-80.8 ± 5.3	10	12.6 ± 0.7	-18.5 ± 6.3	-151.0 ± 25.0	-32.2 ± 5.9	1.2 ± 0.7	860 ± 152	0,7
**9**	-60.6 ± 25.1	4	10.5 ± 0.7	-23.4 ± 9.9	-75.9 ± 55.3	-25.2 ± 6.0	31.8 ± 20.0	857 ± 314	1,6

Supplementary Table S5. Bacterial strains and plasmids used in this study

Strain or Plasmid	Characteristics	Source or reference
**Strains**		
ATCC14028	*Salmonella enterica* serovar Typhimurium	American Type Culture Collection
VFC278	Pseudomonas aeruginosa CIP104116	Institut Pasteur Collection
BL21 (DE3)	*fhuA2 [lon] ompT gal (λ DE3) [dcm] ΔhsdS λ DE3 = λ sBamHIo ΔEcoRI-B int::(lacI::PlacUV5::T7 gene1) i21 Δnin5*	Merck-Millipore
DHT1	F^- glnV44[AS] recA1 endA1 gyrA96 [Nal^r] thi1 hsdR17 spoT1 rfbD1 cya-854 ilv 691::Tn10	47
VF8158	ATCC14028 Δ*rpoS*Δ*cm*	7
VFD416	ATCC14028 Δ*crl*::*tet*RA	18
VF8098	ATCC14028 Δ*crl*::*cm*	7
VFC326	ATCC14028 Δ*rpoS*::*tet*RA	35
VFC331	ATCC14028 Δ*rpoS*	35
VFD462	ATCC14028 $rpoS_{PA}$	This study
VFD561	ATCC14028 $\Delta rpoS_{PA}$::*tet*RA	This study
VFD513	ATCC14028 $rpoS_{PA}$ Δ*crl::cm*	This study
VFD609	ATCC14028 $rpoS_{PA\text{-}L87R}$	This study
VFD430	ATCC14028 $rpoS_{PA\text{-}L87R}$ Δ*crl::cm*	This study
VFD565	ATCC14028 $rpoS_{STM\text{-}R82L}$	This study
VFE173	ATCC14028 $rpoS_{STM\text{-}R82L}$ Δ*crl::cm*	This study
VFD463	ATCC14028 $rpoS_{STM\text{-}R82E}$	This study
VFD514	ATCC14028 $rpoS_{STM\text{-}R82E}$ Δ*crl::cm*	This study
VFD515	ATCC14028 *katN-lacZ*	13
VFD516	ATCC14028 *katN-lacZ* Δ*crl*::cm	13
VFD518	ATCC14028 $rpoS_{PA}$ *katN-lacZ*	This study
VFD521	ATCC14028 $rpoS_{PA}$ Δcrl::cm katN-lacZ	This study
VFE170	ATCC14028 $rpoS_{PA\text{-}L87R}$ *katN-lacZ*	This study
VFE171	ATCC14028 $rpoS_{PA\text{-}L87R}$ Δcrl::cm katN-lacZ	This study

VFE172	ATCC14028 *rpoS*$_{STM-R82L}$ *katN-lacZ*	This study
VFE195	ATCC14028 rpoS$_{STM-R82L}$ Δcrl::cm katN-lacZ	This study
VFD548	ATCC14028 *rpoS*$_{STM-R82E}$ *katN-lacZ*	This study
VFD551	ATCC14028 rpoS$_{STM-R82E}$ Δcrl::cm katN-lacZ	This study
**Plasmids**		
pETM11	Vector with his-tag and TEV cleavable site, used for expression of σ^S_{STM} and Crl$_{STM}$	G. Stier, EMBL Heidelberg
pET-MCN-EAVNH	Vector with his-tag and TEV site used for expression of σ^S_{PA}, σ^S_{PA} L87R and σ^S_{STM} R82L	[48]
pVFD969	pETM11 expressing (his-tag)$_6$-σ^S	[18]
pVFD970	pETM11 expressing (his-tag)$_6$-Crl$_{STM}$	[18]
pVFD818	pETM11 expressing (his-tag)$_6$-σ^S_{PA}	This study
pVFD823	pETM11 expressing (his-tag)$_6$-σ^S_{PA} L87R	This study
pVFE108	pET-M11 expressing (his-tag)$_6$-σ^S_{STM} R82L	This study
pKT25	BACTH vector designed to express a given polypeptide fused in frame at its N-terminal end with T25 fragment; p15 ori; Kmr	[22]
pUT18	BACTH vector designed to express a given polypeptide fused in frame at its C-terminal end with T18 fragment; ColE1 ori; Apr	[22]
pVFA500	pKT25 expressing T25-σ^S_{STM}	[22]
pVFC629	pKT25 expressing T25-σ^S_{PA}	This study
pVFE161	pKT25 expressing T25-σ^S_{PA} H83Y	This study
pVFD410	pKT25 expressing T25-σ^S_{PA} L87R	This study
pVFE162	pKT25 expressing T25-σ^S_{PA} Q89L	This study
pVFE163	pKT25 expressing T25-σ^S_{PA} K90R	This study
pVFE164	pKT25 expressing T25-σ^S_{PA} P93V	This study
pVFD412	pKT25 expressing T25-σ^S_{STM} R82L	This study
pVFD413	pKT25 expressing T25-σ^S_{STM} R82D	This study
pVFD399	pKT25 expressing T25-σ^S_{STM} R82E	This study
pVFA506	pUT18 expressing Crl$_{STM}$-T18	[16]

Supplementary Table S6. Oligonucleotides used in this study

Name	Sequence (5' – 3')	Purpose
Nco-rpoS-PA	GCACTCAAAAAAGAAGGGCCGGA	Construction of plasmid pVFD818
Xho-rpoS-PAr	AAACTCGAGTCACTGGAACAGCGCGTCA CTC	Construction of plasmid pVFD818
RpoSPAD2_3f	GTCCACTTCGCTCGTCGTGCGCAGAAGGG CG	Mutagenesis for construction of plasmids pVFD823 and pVFD410
rpoSPAD2_3r	CGCCCTTCTGCGCACGACGAGCGAAGTG GAC	Mutagenesis for construction of plasmids pVFD823 and pVFD410
rpoSR82Lf	GTCTATTTTGCGCGTTTGGCACTGCGTGG AGAT	Mutagenesis for construction of plasmids pVFE108 and pVFD412
rpoSR82Lr	ATCTCCACGCAGTGCCAAACGCGCAAAA TAGAC	Mutagenesis for construction of plasmids pVFE108 and pVFD412
rpoSPAD2_2f	CCCGAAGAGGAAGTCTATTTCGCTCGTCT GGCG	Mutagenesis for construction of plasmids pVFE161
rpoSPAD2_2r	CGCCAGACGAGCGAAATAGACTTCCTCTT CGGG	Mutagenesis for construction of plasmids pVFE161
rpoSPAD2_4f	TTCGCTCGTCTGGCGTTGAAGGGCGATCC CGCT	Mutagenesis for construction of plasmids pVFE162
rpoSPAD2_4r	AGCGGGATCGCCCTTCAACGCCAGACGA GCGAA	Mutagenesis for construction of plasmids pVFE162
rpoSPAD2_5f	GCTCGTCTGGCGCAGCGTGGCGATCCCGC TGGT	Mutagenesis for construction of plasmids pVFE163
rpoSPAD2_5r	ACCAGCGGGATCGCCACGCTGCGCCAGA CGAGC	Mutagenesis for construction of plasmids pVFE163
rpoSPAD2_6f	CGCAGAAGGGCGATGTGGCTGGTCGGAA GCGG	Mutagenesis for construction of plasmids pVFE164
rpoSPAD2_6r	CCGCTTCCGACCAGCCACATCGCCCTTCT GCG	Mutagenesis for construction of plasmids pVFE164
rpoSR82Df	GTCTATTTTGCGCGTGACGCACTGCGTGG AGAT	Mutagenesis for construction of plasmids pVFD413
rpoSR82DR	ATCTCCACGCAGTGCGTCACGCGCAAAAT AGAC	Mutagenesis for construction of plasmids pVFD413
rpoSR82Ef	GTCTATTTTGCGCGTGAAGCACTGCGTGG AGAT	Mutagenesis for construction of plasmids pVFD399
rpoSR82Er	ATCTCCACGCAGTGCTTCACGCGCAAAAT AGAC	Mutagenesis for construction of plasmids pVFD399
RpoS-STM-PA-Fw	GACTTGCTAGTTCCGTCAAGGGATCACGG GTAGGAGCCACCTTATGGCACTCAAAAA AGAAGGGCCGGAGTT	Construction of strain D462
RpoS-STM-PA-Rv	AAGGCCAGTCGACAGACTGGCCTTTTTTT GACAAGGGTACTCACTGGAACAGCGCGT CACTCGACA	Construction of strain D462

RpoSSTM-Fw	GACTTGCTAGTTCCGTCAAGGGATCACGGGTAGGAGCCACCTTATGAGTCAGAATACGCTGAAAGTTCA	Construction of strains D463, D432, D565
RpoSSTM-Rv	AAGGCCAGTCGACAGACTGGCCTTTTTTGACAAGGGTACTTACTCGCGGAACAGCGCTTCGATAT	Construction of strains D463, D432, D565
STMrpoSPA-tetFw	GCCCCTGTTGACGCCCGAAGAGGAAGTCCACTTCGCTCGTTTAAGACCCACTTTCACATT	Construction of strain VFD561
STMrpoSPA-tetRv	GATCATCCGCTTCCGACCAGCGGGATCGCCCTTCTGCGCCAGCTAAGCACTTGTCTCCTG	Construction of strain VFD561
RpoSPA-L87R-Fw	TCGCCCCTGTTGACGCCCGAAGAGGAAGTCCACTTCGCTCGTCGTGCGCAGAAGG	Construction of strain VFD609
RpoSPA-L87R-Rv	GATCATCCGCTTCCGACCAGCGGGATCGCCCTTCTGCGCACGACGAGCGAAGTGG	Construction of strain VFD609

Supplementary Methods

DNA manipulations

Standard molecular biology techniques were used[7,29]. Oligonucleotides were obtained from Sigma-Aldrich and are listed in Supplementary Table S6. DNA sequencing was performed by Institut Cochin (Paris-France). Derivatives of plasmids pKT25, used in the BACTH assays, were constructed by cloning PCR-amplified DNA fragments encoding σ^S from *P. aeruginosa* between the the XbaI and KpnI sites of pKT25, as previously described (Supplementary Tables S5 and S6)[22]. Plasmid pVFD818 was obtained by cloning PCR-amplified DNA fragment from total DNA of *P. aeruginosa* between the NcoI and XhoI sites of pETM11. Single point mutagenesis of plasmids pVFD818 and pVFD969 (to obtain plasmids pVFD823, pVFE108, respectively) and pVFC629 (to obtain plasmids pVFE161, pVFD410, pVFE162, pVFE163, pVFE164, pVFD412 pVFD413 and pVFD399) were performed using a mixture of DNA template (35 ng), dNTP (25 mM), enzyme Phu turbo (Agilent) and the oligonucleotides listed in Table S5 (10 µM). After PCR reaction, the mixtures were digested by DpnI enzyme (Biolabs). All plasmids were confirmed to be correct by DNA sequencing.

Electrophoresis and immunoblot analysis of proteins

Whole-cell extracts were prepared and SDS-polyacrylamide gel electrophoresis was carried out as described[49]. The amount of protein in whole-cell lysates was determined using the DC Protein Assay kit (Bio-Rad). Equal amounts of protein were loaded in each slot. The molecular weights of the proteins were estimated using Precision Plus Protein Standard (Bio-Rad). Proteins were transferred to PVDF membranes (Hybond P membranes, GE Healthcare) and incubated with the antibody as previously described. Rabbit antibodies against $\sigma^S{}_{STM}$ and Crl_{STM} were from Coynault *et al.*[50] and Monteil *et al.*[7], respectively. Rabbit antibodies against T25 were kindly supplied by Dr. Daniel Ladant from Institut Pasteur. Bound antibodies were detected using secondary anti-rabbit (NA934, GE Healthcare) antibody linked to peroxidase and the Pierce ECL2 Western blotting substrate (Thermoscientific).

ITC experiments

ITC experiments were carried out at 25°C using a MicroCal VP-ITC microcalorimeter (GE Healthcare) with a cell volume of 1.5 mL. In each titration experiment, volumes of 7-10 µL of

a solution containing Crl at concentrations of 200-230 µM were injected into a σ^S-containing solution in the same buffer (50 mM Na-phosphate pH 8.0, 300 mM potassium glutamate). σ^S samples were prepared at concentrations of 15-20 µM. The binding isotherms were obtained by plotting the corrected heat values as a function of the Crl:σ^S molar ratio. The resulting isotherms were fitted to a single set of identical sites model using the Origin software supplied with the calorimeter. $\Delta_b H$ (reaction enthalpy change in kJ mol^{-1}), K_b (binding constant in M^{-1}), and n (number of binding sites) were the fitting parameters. The Gibbs energy and the entropic contribution were calculated using the relationships $\Delta_b G = -RT \ln K_b$, (R = 8.314 Jmol^{-1}K^{-1}, T= 298K) and $-T\Delta_b S = \Delta_b G - \Delta_b H$. All experiments were performed in triplicate and standard deviations were calculated.

CD experiments

CD experiments in the far-UV region were performed using an Aviv CD spectrometer model 215 equipped with a water-cooled Peltier unit. Proteins were concentrated at 0.3-0.5 mg/mL in 50 mM sodium phosphate pH 8.0, 300 mM NaCl. Spectra were recorded at 20°C in cells with a path length of 0.1-mm (121.QS, Hellma). Three consecutive scans from each sample were merged to produce an averaged spectrum. Independent spectra were recorded. The spectra were corrected using buffer baselines measured under the same conditions. The molar ellipticity per mean residue, [θ] in deg cm^2 dmol^{-1}, was calculated from the equation $[\theta]=[\theta]_{obs}$ mrw (10 l C)$^{-1}$,where $[\theta]_{obs}$ is the ellipticity measured in degrees, mrw is the mean residue molecular weight (111.5 Da), C is the protein concentration in g/mL, and l is the optical path length of the cell in cm.

Supplementary References

[44] Thompson, J. D., Higgins, D. G. & Gibson, T. J. CLUSTAL W: improving the sensitivity of progressive multiple sequence alignment through sequence weighting, position-specific gap penalties and weight matrix choice. *Nucleic Acids Res* **22**, 4673-80 (1994).

[45] Garcia De La Torre, J., Huertas, M. L. & Carrasco, B. Calculation of hydrodynamic properties of globular proteins from their atomic-level structure. *Biophys J* **78**, 719-30 (2000).

[46] Laskowski, R. A., Rullmannn, J. A., MacArthur, M. W., Kaptein, R. & Thornton, J. M. AQUA and PROCHECK-NMR: programs for checking the quality of protein structures solved by NMR. *J Biomol NMR* **8**, 477-86 (1996).

[47] Karimova, G., Pidoux, J., Ullmann, A. & Ladant, D. A bacterial two-hybrid system based on a reconstituted signal transduction pathway. *Proceedings of the National Academy of Sciences of the United States of America* **95**, 5752-5756 (1998).

[48] Diebold, M. L., Fribourg, S., Koch, M., Metzger, T. & Romier, C. Deciphering correct strategies for multiprotein complex assembly by co-expression: application to complexes as large as the histone octamer. *Journal of structural biology* **175**, 178-188 (2011).

[49] Silhavy, T. J., Berman, M. L. & Ernkvist, L. W. Experiments with Gene Fusions. *New York: Cold Spring Harbor Laboratory Press* (1984).

[50] Coynault, C., Robbe-Saule, V. & Norel, F. Virulence and vaccine potential of *Salmonella typhimurium* mutants deficient in the expression of the RpoS (sigma S) regulon. *Molecular microbiology* **22**, 149-160 (1996).
